# Motif mapping during chickpea germination reveals a complex sequential activation of different proteolytic activities

**DOI:** 10.1371/journal.pone.0307481

**Published:** 2024-10-31

**Authors:** Indrani Bera, Michael O’Sullivan, Caitriona Scaife, Gerard Cagney, Denis C. Shields

**Affiliations:** 1 Conway Institute of Biomolecular and Biomedical Research, UCD, Dublin, Ireland; 2 School of Medicine, UCD, Dublin, Ireland; 3 UCD Institute of Food and Health, School of Agriculture and Food Science, UCD, Dublin, Ireland; 4 School of Biomolecular and Biomedical Science, UCD, Dublin, Ireland; University of California Riverside, UNITED STATES OF AMERICA

## Abstract

Despite the importance of grains and legumes in the human diet, little is known regarding peptide release and the temporal changes of protease activities during seed germination. LC/MS-MS peptidomic analysis of two cultivars of germinating chickpea followed by computational analyses indicated cleavage dominated by proteases with a single position preference (mainly before (P1) or after cleavage (P1’): L at P2 (cysEP-like); R or K at P1 (vignain-like), N or Q at P1 (legumain-like); and previously unidentified K, R, A and S at P1’; A at P2’). While P1 N cleavages were relatively constant, P1’ K/R preferences were high in soaked garbanzo (kabuli) seeds, declined by four days, and returned at six days, but were much rarer in the brown (desi) cultivar. Late Embryogenesis Associated (LEA) peptides were markedly released during early germination. Vicilin peptides rich in glutamic acid near their N-termini markedly increased with germination, consistent with strong proteolytic resistance, even to human digestion, as indicated by analyses of separate datasets. Thus, this first peptidomics study of seed germination proteolytic profiles unveils a complex cultivar-specific programme of sequential activation and inactivation of a series of proteases, associated with the differential release of peptides from different protein groups.

## Introduction

Plant proteins are becoming increasingly important as a means of meeting nutritional requirements while minimising impacts on climate change [1] and improving health such cardiovascular aspects [2]. Plant-based proteins may potentially reduce diet-related land use by 76%, diet-related greenhouse gas emissions by 49%, eutrophication by 49%, and green and blue water use by 21% and 14%, respectively [3]. Legumes contribute strongly to plant-derived protein intake in human diets, particularly among vegetarians and vegans [4]. Among the legumes, chickpea is the most important in the diets of people in African and Asian countries [5]. Chickpeas are high in protein content (~25%), low in fat content, and rich in bioactive compounds like phenols and isoflavones [4]. Along with the presence of nutritional components, chickpeas contain anti-nutritional factors such as protease inhibitors, amylase inhibitors, phytolectins and others [6]. These anti-nutritional factors, specifically protease inhibitors, decrease the nutritional value of chickpeas by hindering the proteolysis of proteins and thereby reducing digestibility. Protease inhibitors can comprise up to 10% of seed protein, and are likely to play key roles in combating pathogens (fungi, bacteria, nematodes and viruses) and insect herbivores [7].

Transitioning to a more plant-based protein in the diet requires the development of foods which are more easily assimilated. In plants, there is large-scale protein turnover associated with germination. Germination of legumes involves extensive changes which impact on digestibility of the protein [8]. Cysteine endopeptidases are physiologically important in breaking down storage proteins during seed germination [9]. They are typically deposited within vacuoles, and prevented from carrying out inappropriate proteolysis by compartmentalisation, and by storage in a proenzyme form [10]. Over 50% of chickpea seed protein is composed of salt soluble globulins, around 10% of water soluble albumins, around 20% of acid/base soluble glutelins and 5% of alcohol/water soluble prolamins [7]. Vicilin (7S) and legumin (11S) comprise a major portion of the globulin and albumin fractions of chickpea [11], with lipoxygenase, convicilin, lectinβ and LEA (late-embryogenesis associated) proteins also being at high abundance in total protein [12]. Various studies have identified the changes in protein and amino acid content, changes in proteolytic activities with changes in protease inhibitors and antinutritional factors during germination [8, 13]. Similarly in cereals protein digestibility and protein content changes have been quantified [14]. However, the process of protein breakdown and peptide release from the different sets of major legume proteins is not well understood.

Peptidomics provides a method to describe peptides released during germination. An elegant *ex vivo* digestion study showed better mammalian digestibility of chickpea sprouts when compared to ungerminated seeds [15], and their peptidomics analysis characterised chickpea peptides resistant to the stomach, duodenal lumen and brush border membrane phases of mammalian digestion [15]. Peptidomics not only describes the peptides released, but also gives insights into the patterns of proteolysis, since some endoproteases have specific cleavage patterns, which will be in evidence at peptide termini. While these patterns have been used to predict enzymes involved in infant human milk digestion [16–18], and to characterise in detail the specificity of a seed protease [19], it has not been used to systematically track the changes in proteolysis occurring during seed germination, which is the aim of this study.

Here, we investigated the pattern of changes in proteolysis in germinating chickpea by monitoring shifts in the terminal preferences of peptides, and in the protein sources of released peptides. This revealed complex differences in the major proteolytic players not only over the time course of germination, but also between cultivars, involving both known and novel endoproteolytic specificities.

## Materials and methods

### Materials

Diethyl ether (99.5% purity), NaCl (>99.5% purity), Pierce Trypsin Mass Spectrometry grade, Iodoacetamide ultra-pure (98% purity) proteomics Grade, TCEP (98% purity), Pierce Water LC-MS Grade, Acetonitrile (99.9%), Trifluoroacetic acid (99.5%) biochemistry grade were procured from VWR chemicals and 0.1% (v/v) formic acid LC-MS grade was procured from Fisher Scientific.

### Methods

**Fig 1:**
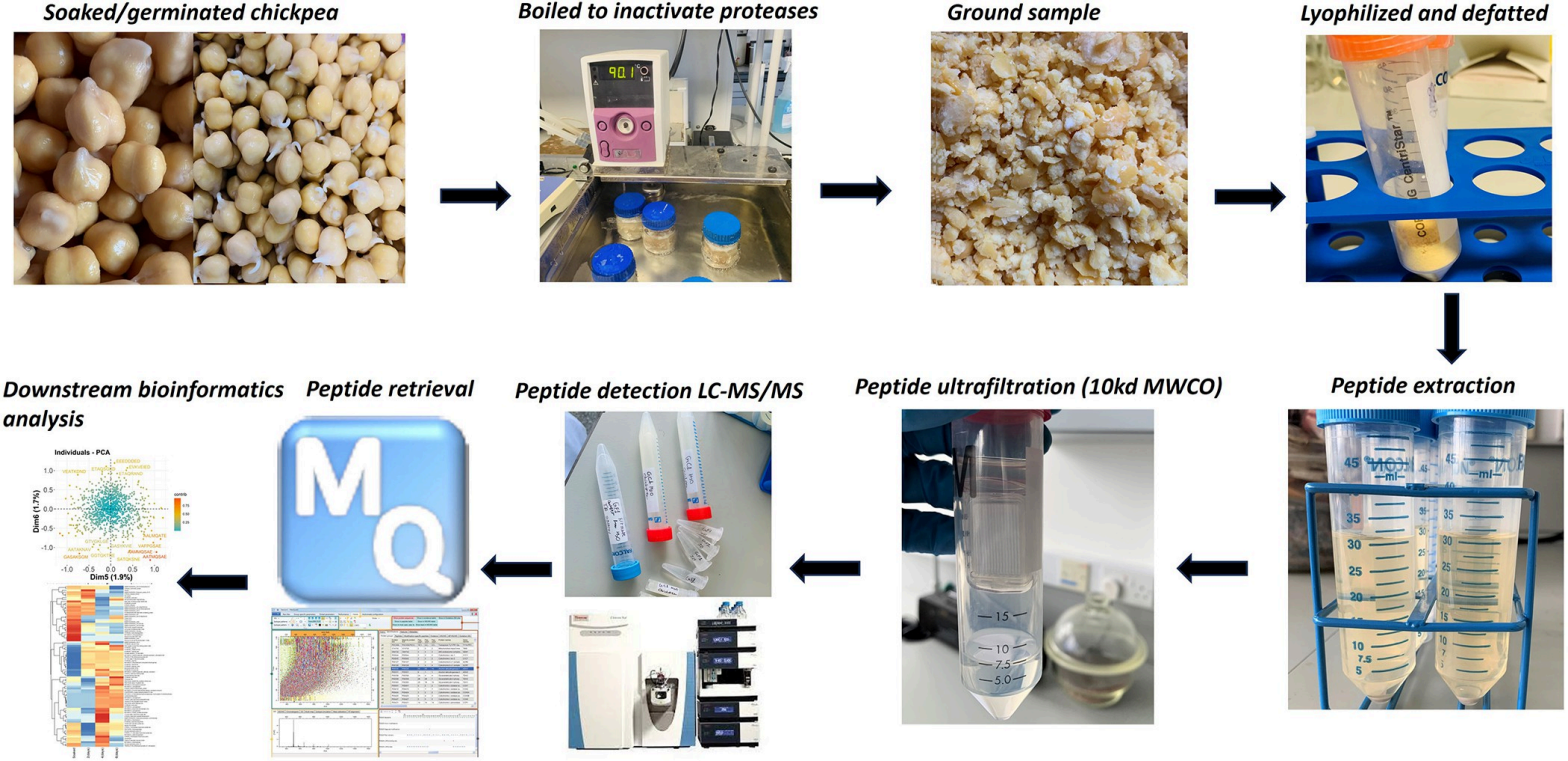
Schematic showing the different steps for peptide extraction and sample preparation of peptidomics.

The methods are described below and summarized schematically in Fig 1.

### Germinating chickpeas

Garbanzo and small brown chickpeas were soaked separately for 12 hours, and then divided into 2 parts in a 30:70 ratio. The part with 30% chickpeas of the initial batch was divided into 3 glass bottles and were used as soaked samples. The remaining 70% were divided into 3 containers and germinated at 18°C. Samples of germinated chickpeas were taken out from each container after 2, 4 and 6 days of germination after 12 hours of soaking in water. Thus 4 times points; 12 hours (soaked), 60 hours (2 days germinated), 108 hours (4 days germinated) and 156 hours (6 days germinated) were considered for further studies. At each time point the samples, in glass jars, were immediately immersed in a water bath and heated to 90°C for 20 minutes to eliminate any further proteolytic activity. Following cooling the seed coats were then removed manually and the dehulled seeds chopped into pieces before freeze-drying and grinding to a fine powder for storage at -20°C before analysis.

### Defatting

To defat the fine chickpea powder, 30ml diethyl ether [20, 21] was added per 5gm sample and shaken for 2 minutes, and then put in an ice bucket for 15 minutes and the supernatant discarded. This was repeated three times. The defatted samples were air dried at room temperature and then stored in 50 mL polypropylene tubes at -20°C.

### Determination of Soluble Nitrogen

The crude protein content was estimated by the Kjeldahl method using the conversion factor of 6.25 [22] on three replicates and protein concentration for each sample is reported as the average of these in Table 1. In brief, 0.5 g of sample was taken into Kjeldahl tubes and a Kjeldahl digestion tablet was added to them. 25 ml concentrated H_2_SO_4_ was added to each of the tube and they were placed in digestion chamber at 200°C for 30 minutes and then at 300°C and 400° C for 30 minutes respectively. The flask was then cooled and 75 mL of water was added carefully. The tubes were placed in distillation unit where ammonia was collected in 25ml boric acid. Distillation was carried out until ~ 150ml of distillate was collected. It was then titrated against 0.1 M HCl.

**Table 1:** % protein (Kjeldahl method) and protein diversity of peptides across germination.

	Average % protein content by dry weight ± SD of three replicates		Number of identified proteins in non-trypsinised sample	
**Time points**	**Garbanzo**	**Brown**	**Garbanzo**	**Brown**
**12 hrs (soaked)**	26.83 ±0.58	21.78 ±0.42	179	115
**60 hrs (2 days)**	28.62 ±0.34	24.84 ±0.45	135	119
**108 hrs (4 days)**	30.12 ±0.88	24.86 ±0.31	240	162
**156 hrs (6 days)**	29.35 ±0.53	24.54 ±0.42	172	149

### Peptide extraction and filtration from non-trypsinised samples

Defatted chickpea powder from all four time points (soaked, 2,4 and 6 days with 3 replicates each) were resuspended in separate aliquots in 0.2M NaCl solution and in distilled water in glass beakers as 10%w/w solutions by dissolving 5gm of chickpea powder in NaCl and, making the total volume to 50ml to extract albumin and globulin peptides [23, 24]. The dispersions were stirred for 2 hours by a magnetic stirrer at room temperature and then placed in the refrigerator overnight. Next day, the extracts containing soluble peptides were collected by placing them in polypropylene tubes and centrifuging at 12,000g for 20 minutes at 20°C. The soluble supernatant (15mL) combined from NaCl and water extraction were transferred very carefully into 10kD MWCO centrifugal filtration module (Amicon Ultra Centrifugal Filter, 10 kDa MWCO) and centrifuged at 5000g for 20 minutes. The collected permeate fractions were stored at -20°C before further processing for injecting into the LC column for LC/MS-MS. Two technical replicates of the mass spectrometry analysis were obtained from each of the biological replicates.

### Preparation of trypsinised samples

For preparing the trypsinised samples, 1ml of soluble peptide extract (supernatant after 12,000 x g centrifugation as described above) was diluted 10 times with HPLC grade water. 50μl of the peptide extract from each sample was retained for digestion. The samples were trypsinised as Pierce manufacturer’s instructions for MS grade trypsin. Each sample was first reduced by incubation for 1 hour at 60°C in the presence of 3mM TCEP (tris(2-carboxyethyl)phosphine, 98% purity)) followed by alkylation by incubation in the dark for 30 minutes at room temperature in the presence of Iodoacetamide (final concentration of 12mM, 98% purity). Trypsin was added in a ratio of 1 μg enzyme to 10μg sample, and samples were incubated overnight at 37°C. The following day the samples were dried on a vacuum evaporator (miVac Duo concentrator, Genevac UK) followed by resuspension in 20μl 0.5% TFA for de-salting using C18 Ziptips (ZTC18S096) according to the manufacturer’s guidelines. Peptides were finally resuspended in 0.1% formic acid prior to loading onto the mass spectrometer.

### Peptide detection using LC-MS/MS

Peptides were characterised using Orbitrap LC-MS/MS. An equivalent amount of each sample (5μl) was injected onto a Dionex Ultimate 3000 (RSLCnano) chromatography system connected to a Thermo Scientific Q Exactive mass spectrometer. Tryptic and non-tryptic peptides from each sample were loaded onto a fused silica emitter (75 μm ID, pulled using a laser puller (Sutter Instruments P2000), packed with Reprocil Pur C18 (3 μm) reverse phase media (Dr. Maisch HPLC GmbH) and was separated by an increasing acetonitrile gradient from 1% Solvent A (0.5% acetic acid, 2.5% acetonitrile) to 30% Solvent B (0.5% acetic acid, 97.5% acetonitrile) over 60 minutes at a flow rate of 250 nL/min. The QExactive MS/MS has an accuracy of less than 3 ppm (external) RMS. The mass spectrometer was operated in data dependent TopN 12 mode, with the following settings: mass/charge (m/z) range 300-1600Th; resolution (m/Δm) for MS1 scan 70,000 at m/z = 200; Automatic Gain Control (AGC) target 3e6; resolution for MS2 scan 17,500 at m/z = 200; AGC target 5e4. The mass spectrometry data have been deposited to the PRIDE Archive (http://www.ebi.ac.uk/pride/archive/) via the PRIDE partner repository (identifier PXD048224).

### Analysis of mass spectra

Raw MS/MS spectra were analyzed in MaxQuant software version 2.0.3.0 [25]. Technical replicates yielded similar total peptide counts, with greater variability in peptide counts among the biological replicates (S1 File). Accordingly, peptide counts obtained from the two technical replicates were pooled for further analysis. The raw files were searched against the *Cicer arietinum* protein database (Uniprot, Downloaded 27/09/2021, 38500 protein entries). Peptides between 6–25 residues were searched for, with variable modifications considered for methionine oxidation and protein N-terminal acetylation. Up to 2 missed cleavages were allowed. The protein false discovery rate (FDR) was set at ≤ 0.01 with a peptide FDR of ≤ 0.01. Match between runs (MBR) and label-free quantification (LFQ) options were selected. MS/MS tolerance was set at 20 ppm. For non-digested samples a nonspecific digestion mode was selected. For digested samples, trypsin was selected as the enzyme, and a fixed modification of carbamidomethylation on cysteine. MaxQuant output files were further analysed. Peptidomic (non-typsinised) analyses were carried out after filtering the peptides based on PEP (Posterior Error Probability) ≤0.001, where PEP is the probability of a false hit. Quantitative analyses were carried out using R [26]. pLogo [27] was used to visualise the enrichment and depletion of amino acids around N and C terminal cleavage sites separately, of the set of unique identified peptides. Letter size is proportional to statistical significance of either enrichment (above the line) or depletion (below the line), with a red line indicating the size of a statistically significant effect. Smaller illegible amino acid letters are typically insignificant. Both peptide counts and log10 of intensities provided approximate relative quantifications of peptides. Peptide and protein distribution across the top 100 identified proteins (defined by peptide count) within each cultivar were tabulated for major functional categories which we defined manually (Fig 5), since gene ontology is underdeveloped for the major protein classes in the chickpea seed.

## Results

### Differentiation between peptide N and C terminal motifs

We investigated the make-up of chickpea seeds at various stages of germination, searching for endogenous peptides (6–25 residues long) by LC-MS/MS. Analysis of trypsinised samples enabled us to understand the protein make-up of the seeds that are the major contributors to the identified peptides, and also to understand functional changes that may arise from changes in protein composition.

After germination, there was a modest increase in protein content as a percentage of dry matter for both garbanzo and brown chickpeas (Table 1), which may reflect both an increase of protein synthesis and a breakdown of carbohydrate and lipid. Between 600 and 1984 endogenous peptides were identified at the four germination stages in the two cultivars, yielding large enough sample sizes to characterise amino acid preferences at peptide termini.

**Fig 2:**
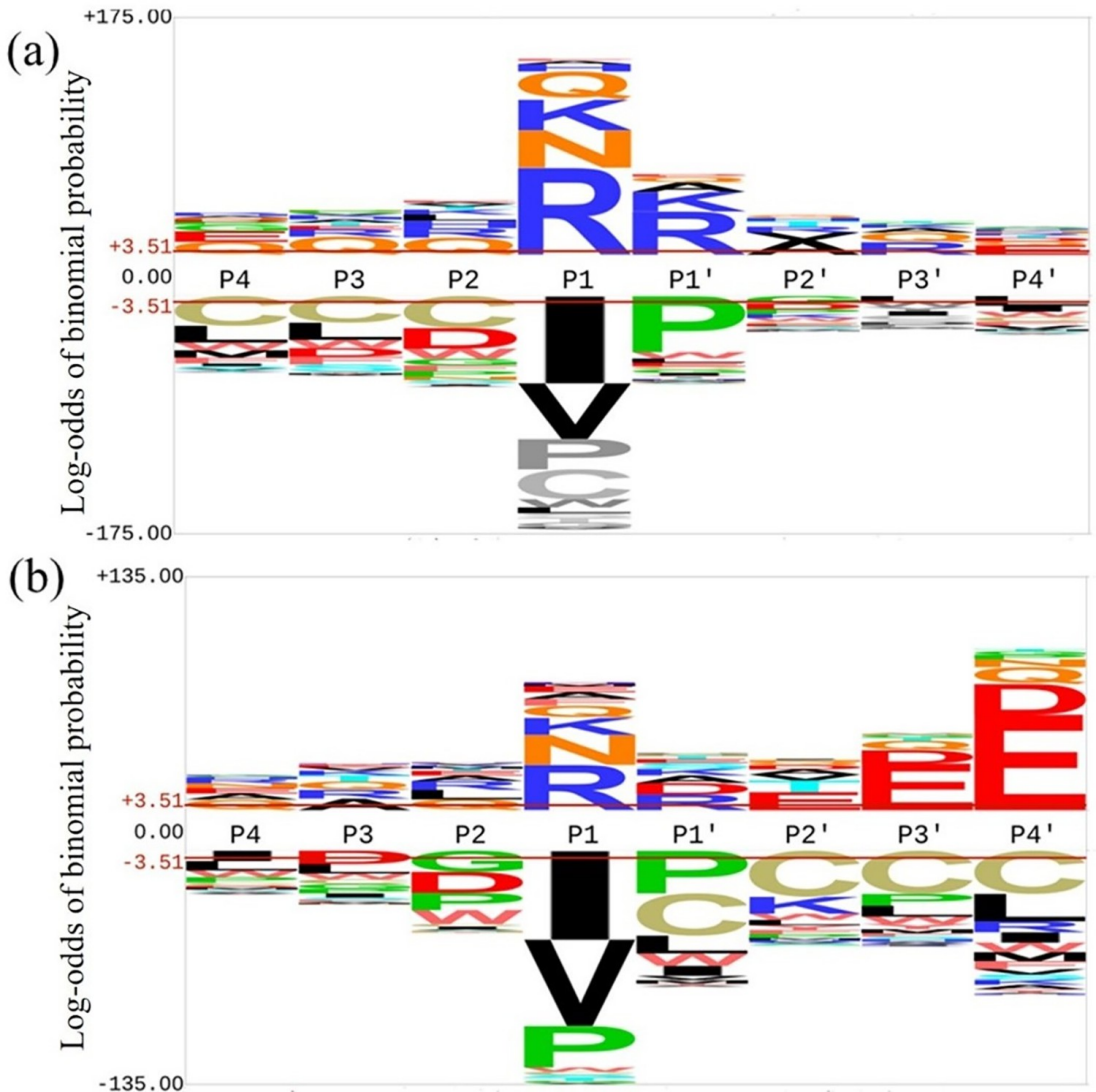
Probability of residue enrichment (above line) or depletion (below line) around peptide cleavage (between positions P1 and P1’ at (a) C-termini and (b) N-termini in a merged peptidomic sample of both cultivars across all germination time points.

To understand the endogenous proteolysis pattern of seed proteins and how they differ as germination progresses, we inspected four residues upstream (termed P4-P1), and four residues downstream of the peptide termini (termed P1’-P4’), since these residues commonly play roles in determining protease specificity. We first investigated the major preferences across the four time points and two cultivars in a single combined analysis. There was an avoidance of cysteines within the actual peptides (at P4-P1 of the C-terminus, and at P1’-P4’ of the N-terminus, Fig 2). A typical consequence of eukaryotic endoprotease specificity is that the strongest amino acid preferences and avoidances are seen at the P1 and P1’ sites, and very similar patterns are seen at both N and C termini (Fig 2). However, N terminal cleavages showed a striking preference for E at P4’ in the N terminal cleavage sites, and a preference for E at P3’ (Fig 2B).

**Fig 3:**
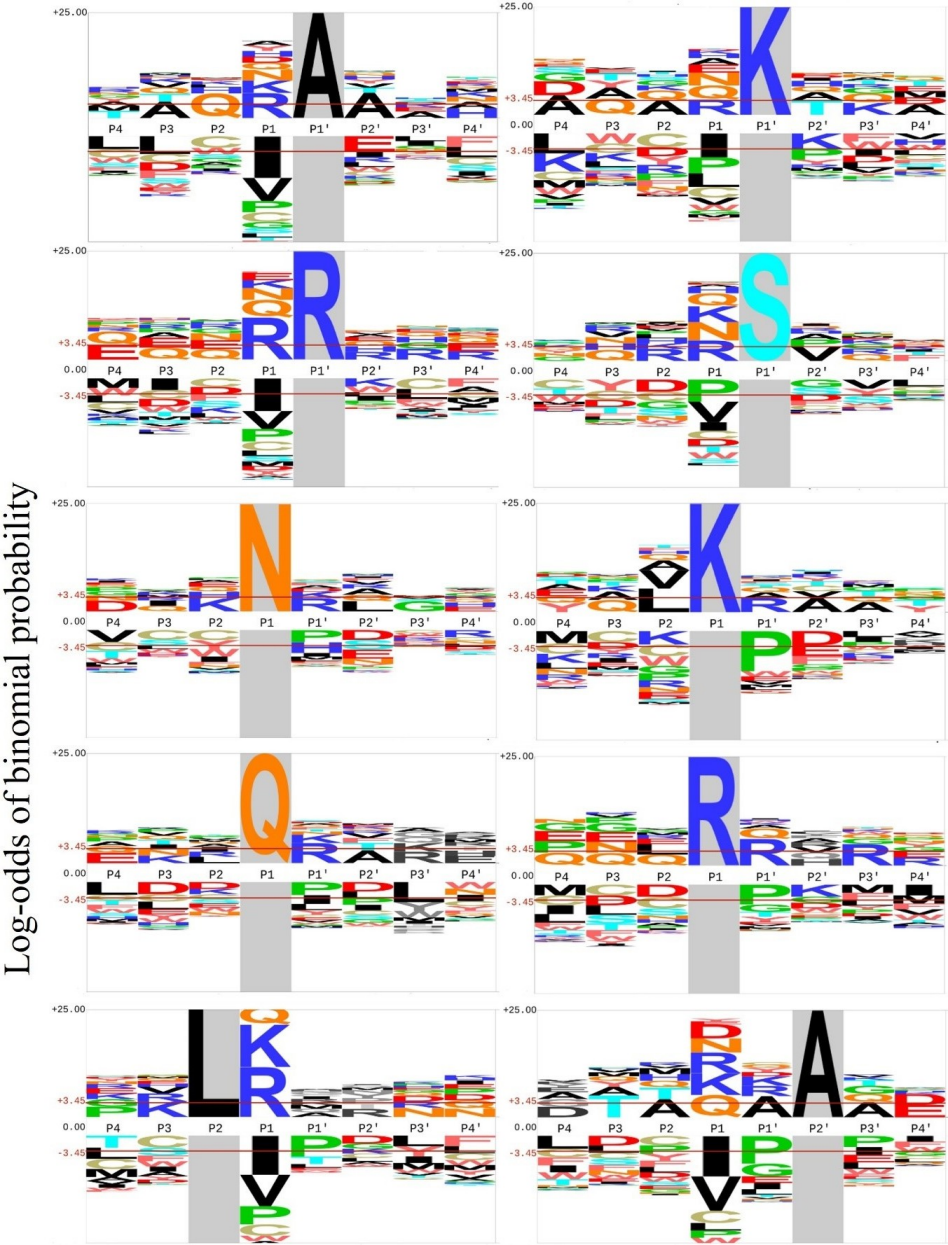
C-terminal P4 to P4’ motif patterns in garbanzo and brown around selected fixed position residues. The most predominant letter is significantly enriched or depleted when it crosses the red line.

Since the pattern of preference for glutamic acid near the N-terminus extended out beyond the region usually playing a role in proteolysis (S1 Fig in S2 File), we considered that this preference likely reflects a selection bias after cleavage in favour of E-enriched peptides arising within the seed or during our biochemical separation of peptides. This contrast between N and C terminal cleavages was even more pronounced in chickpea, both ungerminated and three day germinated, after going through mammalian *in vitro* digestion, from our terminal analysis of data in an independent study [15] (S2 Fig in S2 File). Therefore, we considered the C-termini to provide a better indication of the initial endoproteolysis steps, and focussed subsequent attention on the patterns seen in the C-termini of peptides, which showed stronger amino preferences around the P1 and P1’ sites that most typically play important roles in endoproteolysis. The preferences seen at these sites may reflect motifs involving more than one amino acid, or may reflect simpler motifs dominated by a single residue site (for example, trypsin like). Principal components analysis (PCA) was used to identify a number of enriched motifs involving multiple sites (S3-S5 Figs in S2 File) but the percentage variance accounted for by these was low, with the suggested multi-site motifs, such as an alanine rich motif (S1 Table in S2 File), only accounting for a small number of peptides. Accordingly, we focussed on mapping further the single site preferences over germination, focussing the eight most enriched amino acids in C-terminal peptides (Fig 3), along with P2 Leucine, which accounted for a large number of peptide counts, and P1’ S, suggested from the PCA analysis. The pattern of motif preferences around each of these residues was then inspected (Fig 3 and S3 Table in S2 File).

### Relationship of residue preferences to known endoproteolytic preferences

The preference for P1 N (Fig 2) likely reflects the action of the protease legumain, which is primarily known to cleave after asparagine [28, 29]. While a kidney bean legumain shows a preference for V, F and Y at P3 [28], there is no significant chickpea P3 residue enrichment (Fig 3), perhaps reflecting the diversity of legumain proteases encoded by different genes in plants. Although mammalian legumain shows specificity for N, and D at very low pH [30] we hypothesise that chickpea P1 N and Q cleavage may be mainly performed by legumain or legumain related protease(s), since the original paper that characterised a legumain extract confirmed its ability to cleave after both these residues in a legume [31], and their surrounding profiles of suggestive amino acid preferences are similar (Fig 3).

The preference for cleavage after arginine may reflect the activity of vignain (VPE)-related proteases, which have this activity [31]. Both vignain and legumain are associated with plant vacuoles, which are low-pH and reducing environments [28]. Another plant cysteine protease, mcII-Pa shows a 50 fold preference for arginine over lysine in the P1 position [32]. In addition, there is an apparent serine protease in chickpea that cleaves after arginine [33]. In contrast, the cleavage before R or K in the P1’ is not well mapped to known proteases, although the CPPh/CYSP1-like cysteine protease of kidney bean that digests vicilin efficiently is known to cleave between a serine and lysine [34]. P1’ R prefers R at the P1 position (Fig 3), a preference reciprocated by preferences around P1 R (Fig 3) and suggested by the PCA analysis of garbanzo (S1 Table in S2 File); but this enrichment is not seen for P1’ K, suggesting that they may arise from separate proteases.

The predominance of L at P2 (reminiscent of mammalian cathepsin cysteine proteases [29], resembles that of the castor bean cysteine proteinase enzyme cysEP, whose activity is similar to the vignain-related papain-like enzyme activity accumulating in the legume seed ricinosome which prefers L at P2, with arginine and lysine the preferred amino acids at P1 [35], as seen in Fig 3. This enzyme is associated with destruction of proteins in the endosperm during germination [35]. A more distantly related plant cysteine protease with a more extensively mapped specificity, tobacco NbCP14, has a preference for P2 L>V>I and P1 K>R>G>A [19]. This is consistent with the chickpea P2 L preference for K and R at the P1 site (Fig 3), a preference shared by the mammalian cathepsins [29].

S at P1’ could be associated with a protease resembling stem bromelain, which cleaves within a tri-serine substrate in bromelain inhibitor [36]. P1’ S shows a significant avoidance of proline at P1, suggesting that it may be generated by a distinct enzyme from the endoprotease(s) involved in cleavage before P1’A, which instead shows an avoidance of P1 L. P1’ A and P2’ A show significant enrichment for each-other and similar P1 preferences and avoidances, in particular both strongly avoiding P1 I corroborating with the PCA studies (S1 Table in S2 File).

P1 Isoleucine is the most strongly avoided amino acid at cleavage sites (Fig 2), and this avoidance is distributed across the major subsets of sites (Fig 3). This avoidance may be in common across many cysteine proteases [37]. P1’ S also does not show a significant avoidance of P1 I (Fig 3), suggesting that it may be cleaved by a different class of proteases to the other P1’ -preferring enzymes. P1’ P is strongly avoided by the vignain-like P1 [RK] sites, and also by the legumain like P1 [NQ] sites [38].

There is evidence for an aspartic protease cleaving before aspartic acid during brassica seed development [39], and a legumain-like protease of *Vicia sativa* cleaving after both aspartate and asparagine [10] but we do not see enrichment for cleavage before or after aspartate in chickpea (Fig 2A).

### Changes in proteolytic preferences across germination timepoints and cultivars

**Fig 4:**
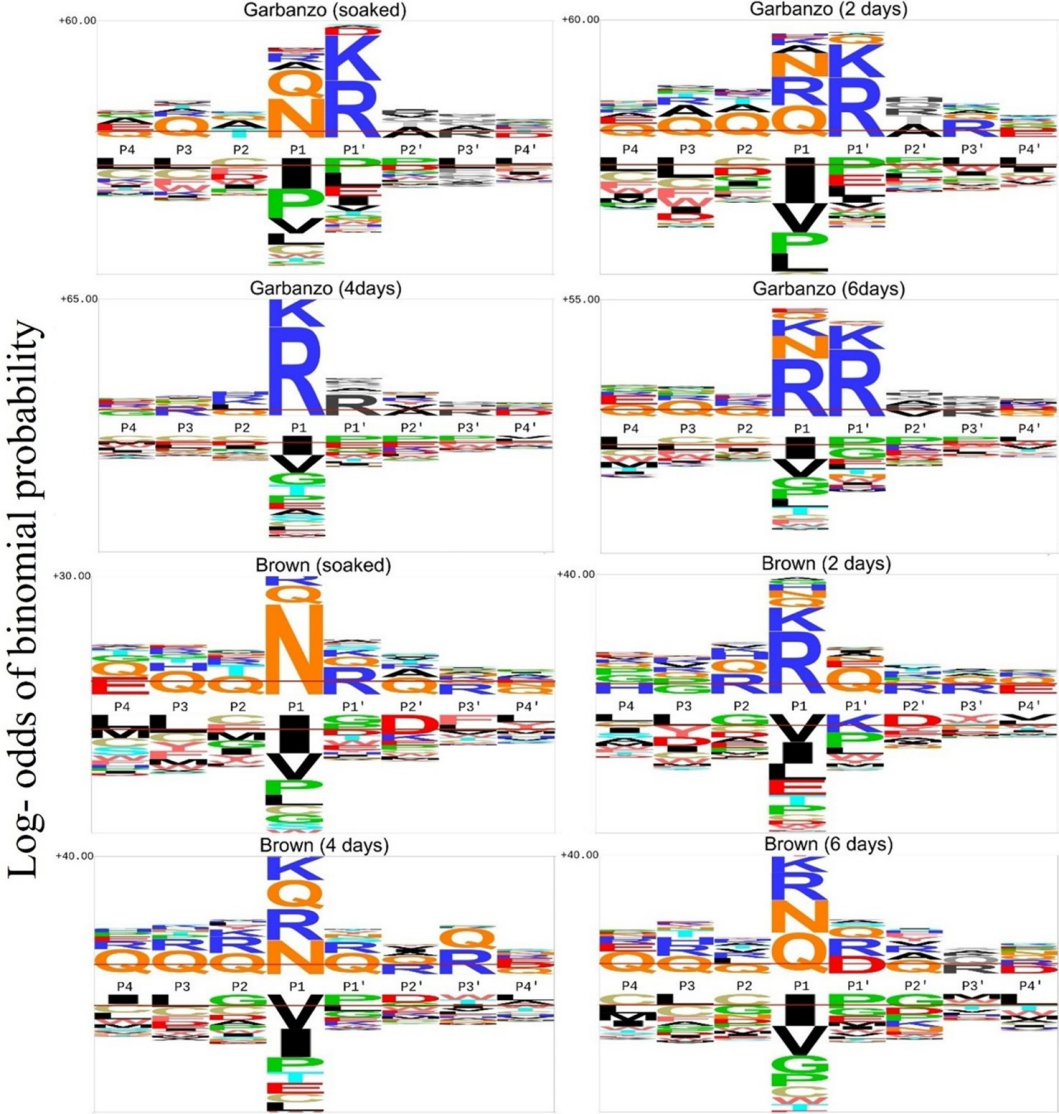
C-terminal motif preferences in garbanzo and brown cleavage sites.

From Fig 4 and Table 2, certain features are shared between the garbanzo and brown cultivars, with overrepresentation of P1 N strongest in soaked versus germinated seeds, and the increase of P1 K and R with germination. In other respects they differ substantially, for example the high frequency of P1’ K and R seen in soaked, 4 day and 6 day garbanzo is largely absent from 2 day garbanzo and from all four stages of brown germination. Avoidance of P1 I and V are seen at all stages in both cultivars.

**Table 2:** Changes during germination in % of amino acids with marked preferences at a particular position. Shades of green and red showing low and high values respectively in garbanzo.

Cleavage position* & Amino acid	% peptides out of total peptide count in garbanzo / brown			
	12 hrs (Soaked)	60 hrs (2 days)	108 hrs (4 days)	156 hrs (6 days)
P2 L *cysEP-like*	6.9 / 6.2	6.5 / 6.6	10.0 / 9.1	6.1 / 9.5
P1 N *legumain-like*	8.7 / 12.2	9.1 / 10.4	11.8 / 14.2	9.4 / 11.7
P1 Q *legumain-like*?	5.6 / 11.1	4.1 / 6.3	4.7 / 11.0	9.2 / 12.1
P1 K *vignain-like*	7.4 / 7.9	10.3 / 11.9	13.8 / 18.1	11.0 / 13.9
P1 R *vignain-like/*	7.3 / 6.5	12.1 / 23.3	24.0 / 11.6	16.9 / 9.5
*mcII-Pa-like*				
P1’ K *cppH-like*?	14.9 / 9.9	14.5 / 1.3	7.5 / 4.5	13.4 / 7.6
P1’ R	15.1 / 11.1	14.5 / 5.0	12.5 / 7.7	18.2 / 8.8
P1’ A	7.6 / 5.7	8.4 / 7.5	9.4 / 6.9	6.9 / 6.7
P1’ S	11.1 / 7.8	11.2 / 7.2	13.7 / 8.8	12.0 / 7.7
P2’ A	11.7 / 10.7	10.4 / 5.1	11.0 / 6.6	9.7 / 8.0
P4’ E*	11.1 / 7.3	27.0 / 10.9	15.1 / 7.6	13.1 / 6.5
**Total peptide count**	1774 / 738	1984 / 604	930 / 1016	1074 / 1257

* C-termini only, except for P4’ E which is N-termini only

The differences in apparent proteolytic signatures seen at each stage seem likely to involve multiple proteases with different specificities dominating the digestion at different stages, but overlapping each-other. In garbanzo C terminal peptides, the positively charged amino acids R and K are preferred at P1’ in soaked, at both P1 and P1’ after two days germination, but at P1 at four days. This is suggestive of one set of proteases being present earlier in germination, overlapping with another protease set which appears to dominate later in germination. This is a very significant effect in garbanzo (S3 Table in S2 File), especially at the C-termini, where at 2 days, cleavage sites are under-represented for side-by-side positive charges at both P1 and P1’ (Odds Ratio 0.47, p = 2x10^-9^), while at 4 days they are strongly over-represented (Odds Ratio 2.01, p = 3x10^-5^). This clearly indicates different proteases with positive charge preferences acting at different times. In contrast, no such enrichments for, or deficits of, two basic residues across the cleavage site were seen in brown chickpea, indicating that the set of most active proteases is very different between the cultivars (S4 Table in S2 File).

### Early release of LEA proteins during germination

**Fig 5:**
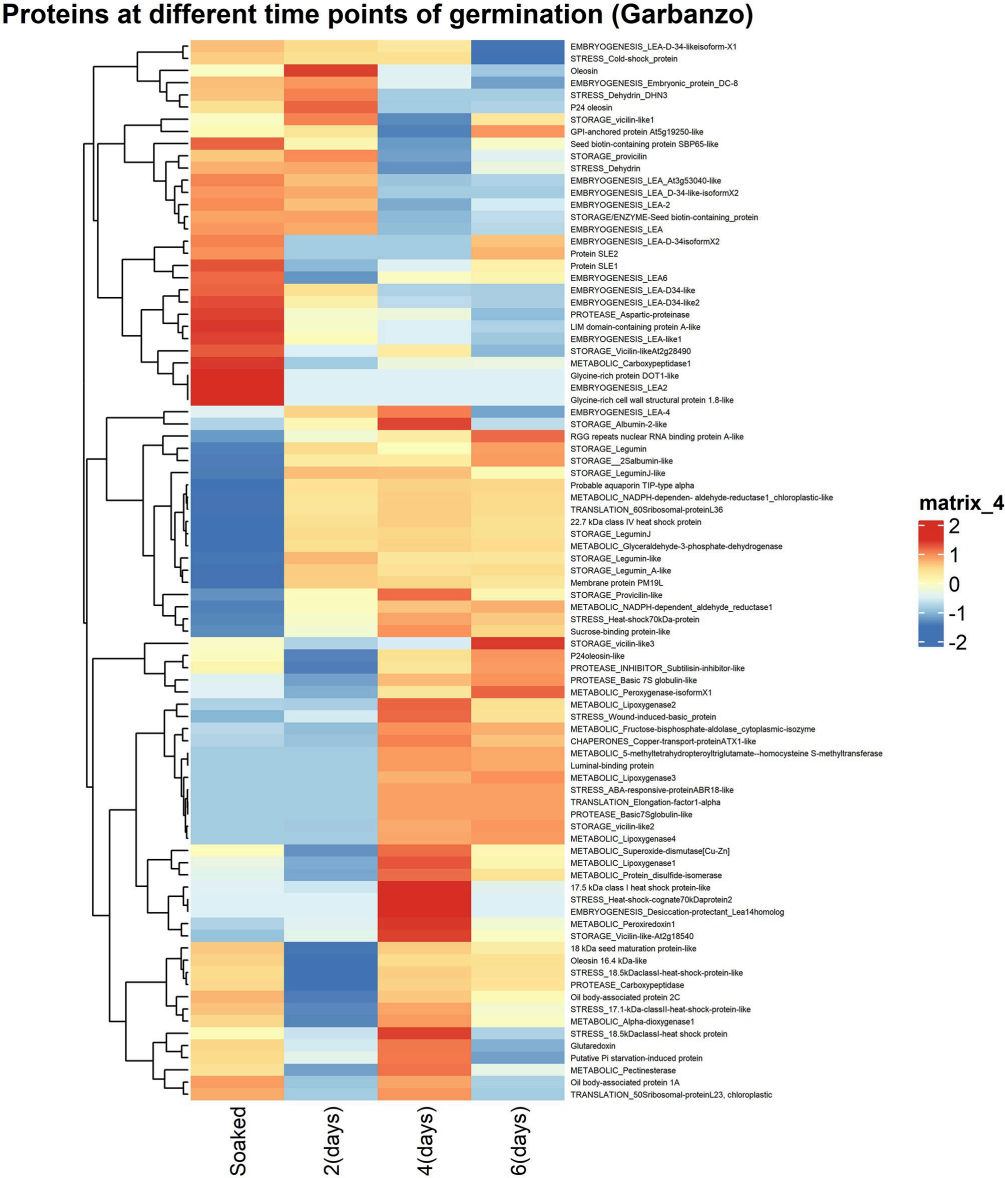
Peptide release from different proteins during germination. Heatmap of z-scores of log10(intensity) of top 100 proteins in non-trypsinised garbanzo. Proteins were assigned to seven major categories indicated in capitals at the start of the protein name.

Gene ontology of the proteins releasing peptides during germination (S7 Fig in S2 File) suggested an increase in proteins with a role in translation during germination. However, the number of proteins with a gene ontology assignment was small, and the total number of peptides accounted for by these proteins was also small. Given the deficiencies in current gene ontology annotation for major legume seed proteins, we classified the top 100 proteins into seven major categories: storage proteins, proteases, protease inhibitors, metabolic, translation, stress and chaperones. These categories are indicated at the start of the names displayed in Fig 5. Other functions commonly seen (Fig 5) included lipid associated proteins. We analysed changes in the proteins observed in both the trypsinised garbanzo (indicative of soluble protein presence) and non-typsinised peptidomic garbanzo and brown chickpea soluble peptide samples (indicative of protein breakdown) (S8 Fig in S2 File).

Peptide counts and percentage of peptides in each group for all the biological sample replicates are tabulated in S6-S8 Tables in S2 File, and summarised in Tables 3–5. Storage proteins release the greatest number of peptides from soaked and germinated seeds, and the number increases up to day six in both cultivars (Tables 3 and 4). However, the trypsinised samples show a modest reduction in the proportion of peptides derived from storage proteins with germination (Table 5), consistent with a gradual depletion of storage proteins over time. The second largest category of embryogenesis proteins contribute to about a fifth of peptides identified throughout germination in the trypsinised samples (Table 5); however embryogenesis peptide release is much more marked early in germination, especially in garbanzo (Tables 3 and 4), dropping from 28% of peptides in soaked to 10% at day 6, indicating that this class of proteins is being most efficiently hydrolysed at the start of germination. While peptide release from metabolic proteins increased over germination in both cultivars (Tables 3 and 4), there was a more complex pattern of metabolic protein representation in the trypsinised samples (Table 5). It may be that some of the metabolic proteins have completed their major function in the germinating seed, and are then being broken down at day 6, so that fewer remain in the trypsinised samples at this stage. We used Fisher’s exact test to measure the statistical significance compared to 12 hours (soaked) samples and significant p-values are indicated.

**Table 3:** Average of % peptides counts for different classes of proteins at different germination time points and significant p-value indicated as compared to soaked sample (*p< 0.05, **p<0.01, ***p<0.001) for garbanzo non-trypsinised with shades of green showing increase and red showing decrease with respect to soaked.

GARBANZO	12 hrs (Soaked)	60 hrs (2 days)	108 hrs (4 days)	156 hrs (6 days)
Storage	44.1	50.81	51.68*	60.53**
Embryogenesis	28.29	22.54	9.796***	9.67***
Protease	1.71	1.14	3.18**	3.01*
Protease inhibitors	0.32	0.23	0.38	0.336
Chaperones	0.07	0.09	0.19**	0.16*
Stress	7.713	8.58	10.57**	8.07
Metabolic	6.05	5.45	14.69***	9.66*

**Table 4:** Average % peptides counts for different classes of proteins at different germination time points and significant p-value indicated as compared to soaked sample (*p< 0.05, **p<0.01, ***p<0.001) for brown non-trypsinised with shades of green showing increase and red showing decrease with respect to soaked.

BROWN	12 hrs (Soaked)	60 hrs (2 days)	108 hrs (4 days)	156 hrs (6 days)
Storage	57.34	55.78	62.30*	68.88*
Embryogenesis	21.03	19.05	11.13***	9.61***
Protease	2.2	0.68***	2.55	2.76**
Protease inhibitors	0.70	0.34*	1.37***	1.03
Stress	5.52	4.59*	4.66*	3.39***
Metabolic	6.94	8.30**	11.82***	10.41**
Translation	0.068	0.29**	0.07	0.05

**Table 5:** Average of % peptides counts for different classes of proteins at different germination time points and significant p-value indicated as compared to soaked sample (*p< 0.05, **p<0.01, ***p<0.001) for garbanzo trypsinised with shades of green showing increase and red showing decrease with respect to soaked.

GARBANZO	12 hrs (Soaked)	60 hrs (2 days)	108 hrs (4 days)	156 hrs (6 days)
Storage	42.47	38.70*	36.30***	39.81**
Embryogenesis	20.31	20.75	18.38*	22.37*
Protease inhibitors	1.18	0.82	0.83	1.03
Chaperones	2.31	2.08	2.92*	2.46
Stress	3.38	4.68**	5.47***	5.32**
Metabolic	7.71	9.85*	12.37**	5.71*
Translation	1.26	1.78*	2.01*	1.23

Table 3 shows that both cultivars release more embryogenesis peptides early in germination, but that the effect is far more pronounced in garbanzo than in the brown chickpea, which has fewer embryogenesis related proteins released overall. *A priori* we would have expected storage proteins to be gradually depleted as a proportion of released peptides over germination, but the timescale of germination studied may be too short for that in the chickpea, where there may well be substantial remaining storage protein in the original seed at day 6. The relative numbers of peptides of the defined classes show a number of pronounced shifts (Table 3) that cannot be simply explained, suggesting that the dynamics of peptide release from each class is influenced by multiple factors that vary with both time point and cultivar.

### Changes in detected proteases and protease inhibitors

We tracked the peptide counts of proteases and their inhibitors from both the tryptic and non-tryptic samples. For proteases, very few have high peptide counts in the tryptic sample (S10 Table in S2 File), which may be because there is such a high preponderance of trypsin-digested storage proteins masking their detection. The most detectable putative protease was “Putative Pi starvation-induced protein” but its functional role in seeds is unknown. In the non-tryptic garbanzo sample (S8 Table in S2 File), peptides derived from aspartic protease and aspartic protease-like proteins are present throughout germination, while legumain appears at higher counts in soaked seeds and tails off during germination matching the decline in its cleavage motif (after asparagine) later in germination in both garbanzo and brown (Fig 4). Thus, while peptide counts in non-tryptic samples are an indicator of protein breakdown, they may be an indirect indicator of soluble protein concentration, although the numbers are too small to provide any reasonable quantification. Changes in protease inhibitor concentrations could also have effects on proteolysis. From the tryptic samples, the three highly detectable inhibitors Bowman-Birk type proteinase inhibitor-like, subtilisin inhibitor-like and 7S globulin-like protease inhibitors showed relatively constant levels throughout soaking and germination. Interestingly, in the non-tryptic samples, the protease inhibitors were not detectable at such high peptide counts (S8 and S9 Tables in S2 File). Together, these two observations are consistent with a relatively high stability of protease inhibitors during germination, so that they may only come to be broken down beyond day 6 of germination.

## Discussion

Despite the importance of grains and legumes in the human diet, there is only patchy knowledge regarding peptide release and the temporal changes of protease activities during seed germination. We took a peptidomics approach to characterise the cleavage preferences across germination in two cultivars of chickpea. Our analysis identified a number of different endoprotease cleavage preferences, most of which were dominated by a single residue preference. Some of these we could partly align with the known cleavage preferences of known plant cysteine proteases (legumain-like P1[QN], vignain-like P1[RK], cysEP-like P2[L]), and other preferences which were difficult to map to likely proteases (K, R, A and S at P1’; A at P2’). A limitation of this study is that the proteases underpinning the cleavages are only suggestively mapped to the preferences, based on the sparse and patchy literature across a variety of legumes and other plants. Future systematic analysis of specificity by proteomics analysis of cleavage of substrates with purified enzymes [40] will in future help map out specificities more convincingly. However, the precise pH and hydration conditions in different compartments of the germinating seed may be difficult to represent *in vitro*. Accordingly, more extensive systematic analysis of combined cleavage of natural seed substrates *in situ* along with more detailed assessment of protease variation across multiple strains and conditions, can in parallel shed light on physiologically relevant protease activities.

The degree of divergence between the endoproteolytic profiles of the brown and garbanzo chickpeas was somewhat surprising. While P1 N (legumain-like) cleavages were relatively constant, the P1’ K/R preferences were high in soaked garbanzo seeds, declined by four days, and returned at six days, but overall these were much rarer in the brown cultivar. This raises very interesting questions, as to why there should be such differences, since presumably they will impact on the overall efficiency of storage protein release, impacting on seedling viability and competition. While the cultivars appear to have roughly similar distribution of proteins by 1D gel electrophoresis, they do have some biophysical differences [41], so it is possible that the proteolytic programme is optimised to differences in their storage protein substrates. However, in one study, compared to garbanzo (kabuli), brown chickpea (desi) had higher levels of both trypsin and chymotrypsin inhibitory activity, and the presence of protease inhibitors is likely to impact differentially on the suppression of certain proteases during germination [42]. Variation in protease inhibitor levels may not only reflect inherent strain differences, but may also reflect environmental differences, since exposure to a major insect pathogen of chickpea, the podborer (*Helicoverpa armigera*), can result in elevated expression of protease inhibitors [36]. Given that the podborer appears to have acquired the ability to proteolytically inactivate protease inhibitors in chickpea [43], it is possible to envisage very strong selection pressures on the proteolysis system in legumes and grains, where protease inhibitors whose primary function may be to inhibit pathogen or insect digestion may in turn impact on the ability of the endogenous proteases to efficiently break down the seed storage proteins.

Peptides from Late Embryogenesis Associated (LEA) proteins were markedly released during early germination, in contrast with peptides from major seed storage proteins, which increased as a proportion of the peptides released later in germination. LEAs represent an important set of under-studied chickpea seed proteins. They are compositionally highly biased, being particularly glycine rich. 22–24 kD LEA proteins accumulate during the late stage of chickpea seed formation [12]. They are likely to perform multi-functional roles, including tolerance to dehydration and cold stress, maintaining space in the mature dessicated seed, acting as membrane and protein protectors, and acting as potential anti-oxidants [44]. Thus, LEA proteins may well have multiple potential effects on yield under drought conditions, on storage stability and on vigorous germination. A key feature of these proteins is their high protein disorder [44, 45], in contrast with the more structured elements of many seed storage proteins. Thus, their rapid mobilisation during early germination may simply reflect the ease with which proteases can digest them, since neither the full proteins nor their partial digestion products are likely to form aggregates or shield hydrophobic cores from cleavage, in a well hydrated environment. For brown chickpea there was a similar, but less marked trend of early LEA peptide release during germination, indicating likely variability in cultivars with respect to these proteins or their proteolysis. Overall, their early release during germination may suggest that the primary functional role of LEA proteins is in the dormant seed, rather than in the sprout.

We noted a difference between cleavage patterns at the N and C termini of peptides, with an enrichment for glutamic acid centred around the P4’ site of N-termini that was absent from C termini (S6 Fig in S2 File). This could potentially reflect exoprotease specificity patterns, such as those of tripeptidyl aminopeptidases [46] which are abundant in seeds of the legume sesame [47]. However, given their greater representation in a separate study [15] (S2 Fig in S2 File) which subjected ungerminated and germinated chickpea to mammalian in vitro digestion (including stomach, pancreatic fluid and brush border membrane proteolysis phases), it appears that these peptides are resistant to a wide variety of proteolysis. We hypothesise that they may have undergone a modification conferring protease resistance that does not alter the overall peptide mass, such as stereo conversion of residues from L- to D- amino acids.

The peptides identified here do not include shorter di- and tri-peptides. However, it is worth noting that a study by LC-MS/MS of soybean oligopeptides [48] showed a preponderance of arginine and lysine at the termini of tripeptides that changed during germination, suggestive of their C-terminal cleavage being performed by a vignain-like protease.

Major classes of peptides identified here that are released during germination include transient LEA-protein derived peptides, and persistent, protease-resistant glutamate rich vicilin-derived peptides. It will be of interest to explore if there are any consequences for gut comfort or health arising from ingesting these peptides in the hydrolysed form generated by seed germination. The proteolysis of seed storage proteins during germination may improve digestibility in the human diet, and breakdown of certain peptides and proteins may also reduce allergenicity [49–51]. Future studies of such health implications should pay careful attention to variation within and between cultivars, some of which is likely to reflect their different growth environments and pathogen exposures.

## Supporting information

S1 File S1 File which contains 5 excel sheets containing protein list along with sequence coverages at different stages of germination and peptide files from MaxQuant output, namely: a) Peptides_Non-trypsinised brown, b) Peptides_Non-tryp Garbanzo, c) Peptides_Trypsinised Garbanzo, d) Proteins trypsinised garbanzo, e) Proteins non-trypsinised, f) Proteins non-trypsinised brown. (XLSX)

S2 File S1-S10 Tables and S1-S8 Fig contain details of information regarding peptide counts, cleavage site preferences and protein expression during different stages of germination. (DOCX)

S3 File (JPG)
