# Supplementary material for: Motif mapping during chickpea germination reveals a complex sequential activation of different proteolytic activities
Source: PLoS One. 2024 Oct 31;19(10):e0307481. doi: 10.1371/journal.pone.0307481 (PMC11527212; doi:10.1371/journal.pone.0307481)
Supplement: S2 File — (DOCX) [file pone.0307481.s002.docx]

**Supplementary Information**

**Motif mapping during chickpea germination reveals a complex sequential activation of different proteolytic activities.**

Indrani Bera^1,2*^, Michael O’Sullivan^4^, Caitriona Scaife^1^, Gerard Cagney^1,3^, Denis C. Shields^1,2*^

^1^Conway Institute of Biomolecular and Biomedical Research, UCD, Dublin, Ireland

^2^School of Medicine, UCD, Dublin, Ireland

^3^School of Biomolecular and Biomedical Science, UCD Dublin, Ireland

^4^UCD Institute of Food and Health, School of Agriculture and Food Science, UCD, Dublin

*Corresponding Authors Emails: [denis.shields@ucd.ie](mailto:denis.shields@ucd.ie), [indrani.bera@ucd.ie](mailto:indrani.bera@ucd.ie)


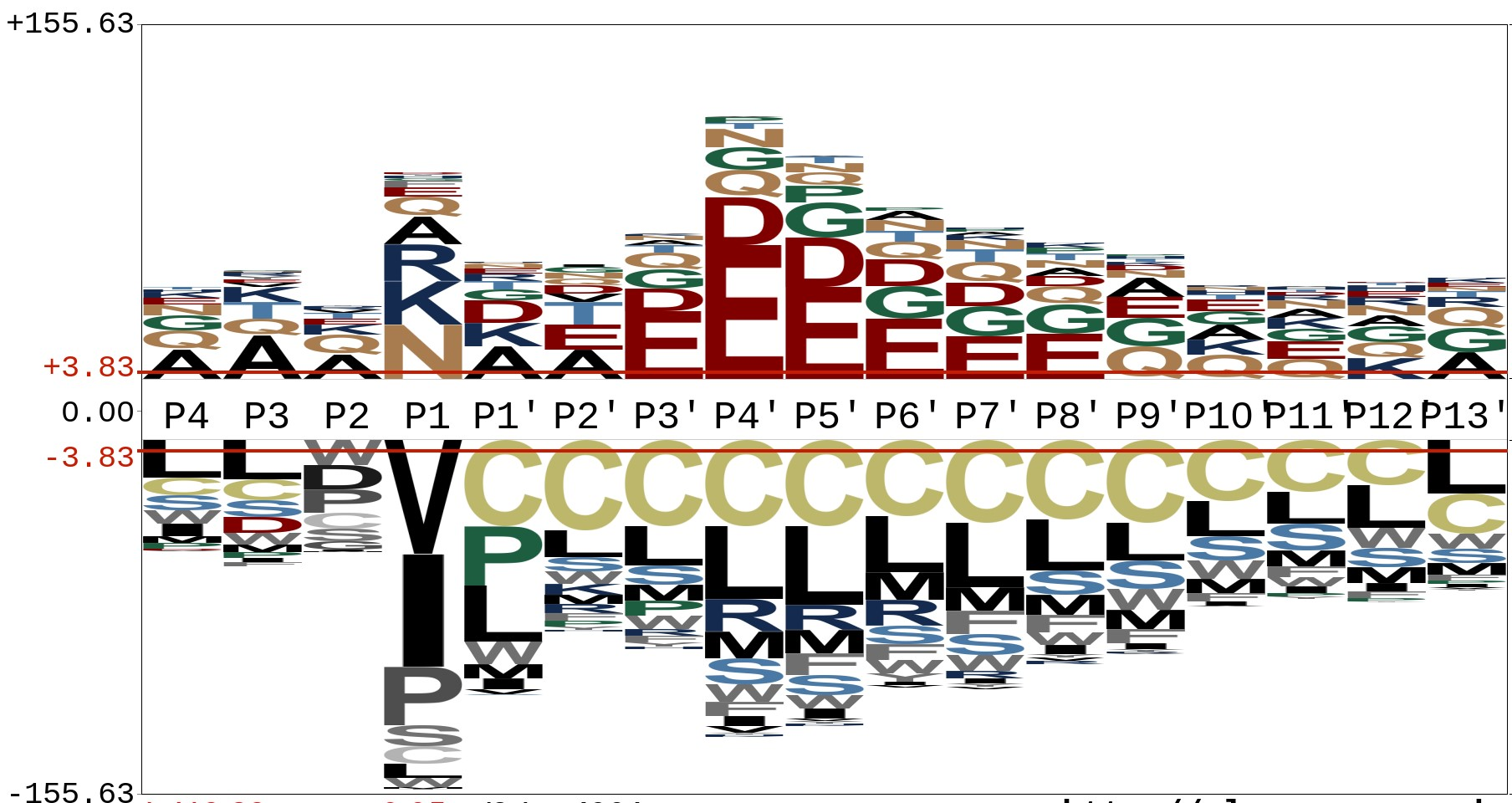


**Figure S1.** Sequence logo for P4 (-4) to P12” (+12) positions with the cleavage site between P1 and P1’


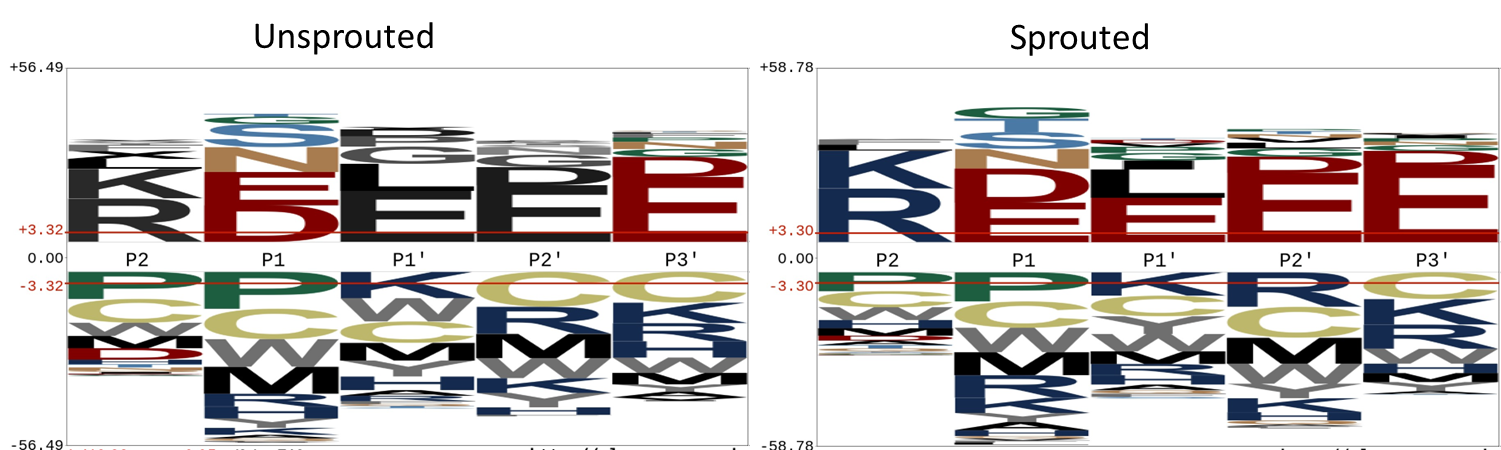


**Figure S2.** Sequence logos N-terminal end of unsprouted and sprouted chickpea peptides found after duodenal digestion.


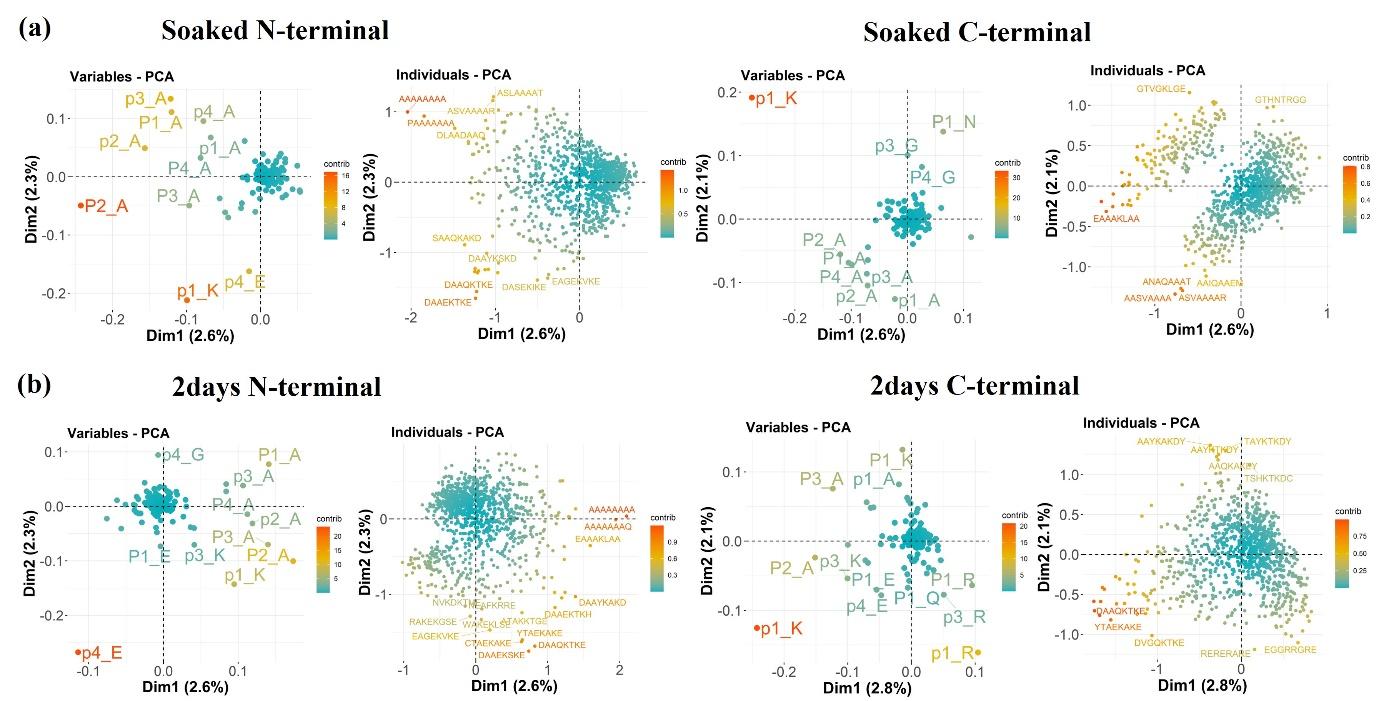


**Figure S3.** PCA plots showing amalgamation of distinct proteases preferring different substrates in garbanzo chickpea (a) soaked (b) 2 days samples


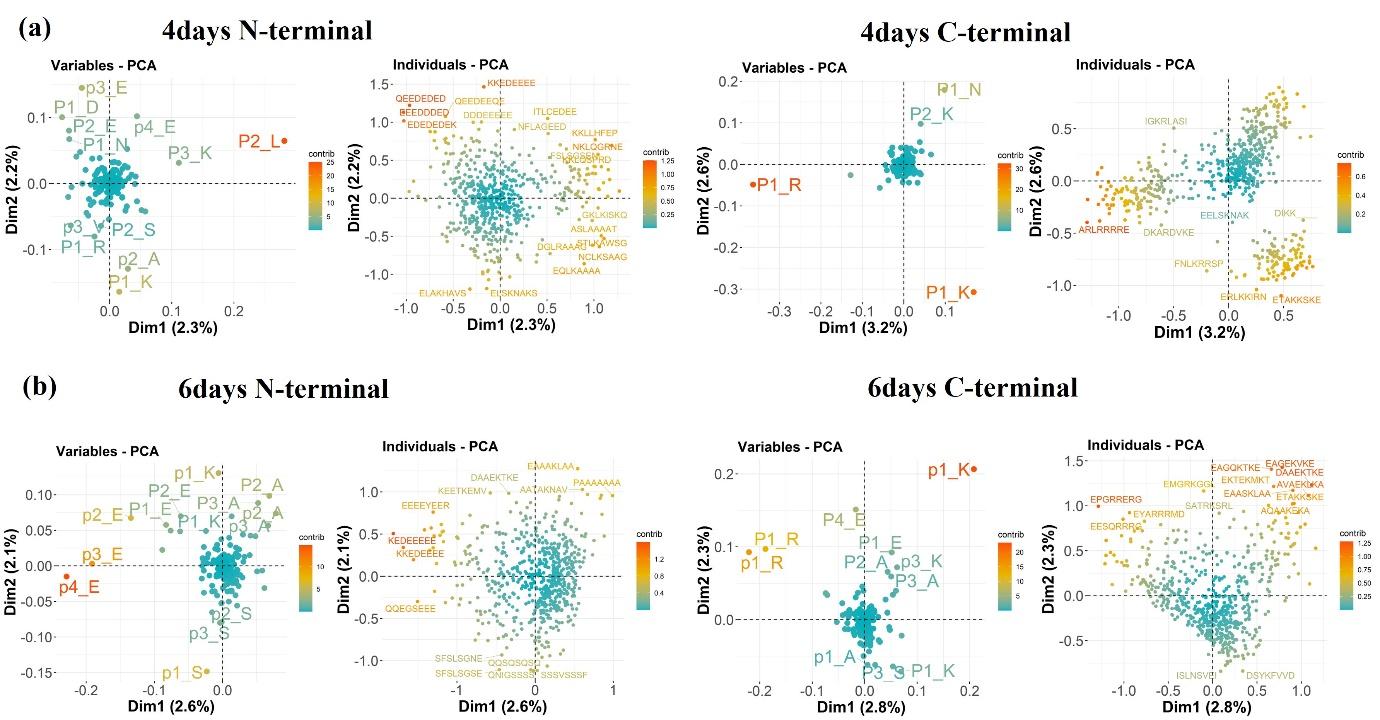


**Figure S4.** PCA plots showing amalgamation of distinct proteases preferring different substrates in garbanzo chickpea (a) 4 days and (b) 6 days samples

**Table S1:** Additional proteolytic site preferences in garbanzo chickpea suggested from the principal component analysis represented as percentage of occurrence for N-terminal and C-terminal. Motifs are highlighted in bold. All numbers calculated as percentage of occurrence (no. of occurrence/no.of peptides)*100

| P4-P3-P2-P1-p1-p2-p3-p4 | Ungerminated  (N-term/C-term) | 2days  (N-term/C-term) | 4days  (N-term/C-term) | 6days  (N-term/C-term) |
| --- | --- | --- | --- | --- |
| X-X-X-A-A-A-X-X | 1.0/0.39 | 0.4/0.40 | 0/0 | 0.1/0.18 |
| X-X-X-X-K-X-X-E | 2.3/1.9 | 2.1/1.91 | 1.2/1.5 | 1.4/1.8 |
| X-X-X-N-X-X-G-X | 0.95/1.3 | 1.2/1.15 | 0.3/1.2 | 0.5/1.0 |
| X-X-X-A-X-X-A-X | 2.4/1.46 | 1.9/2.0 | 1.0/0.21 | 1.1/0.74 |
| X-A-A-X-K-X-X-X | 1.0/0.39 | 0.9/0.65 | 0.4/0 | 0.7/0.27 |
| X-X-X-X-R-X-R-X | 0.1/1.97 | 0.2/1.86 | 0.3/1.7 | 0/2.79 |
| X-A-X-K-A-X-X-X | 0.1/0.05 | 0.3/0.55 | 0.1/0.20 | 0/0.18 |
| X-K-L-X-X-X-X-X | 0.73/0.45 | 0.6/0.85 | 2.4/1.1 | 1/0.74 |
| X-X-X-D-X-X-E-X | 1.0/0.11 | 1.6/0.30 | 1.9/0.21 | 1/0.18 |
| X-X-X-N-X-X-X-X | 8.7/12.2 | 9.1/10.4 | 11.8/14.2 | 9.4/11.7 |
| X-X-X-K-X-X-X-X | 7.4/7.9 | 10.3/11.94 | 13.8/18.1 | 11/13.9 |
| X-X-X-X-S-X-X-X | 11.1/7.8 | 11.2/7.2 | 13.7/8.8 | 12/7.7 |
| X-X-X-R-R-X-X-X | 0.5/1.91 | 0.8/2.3 | 2.2/6.02 | 1.3/4.6 |
| X-X-X-X-K-X-X-X | 13.0/14.9 | 11.3/14.4 | 8.6/7.52 | 11/13.4 |
| X-X-K-X-A-X-X-X | 0.2/0.33 | 0.50/0.30 | 0.2/1.29 | 0.3/1.02 |
| X-X-X-X-X-E-E-E | 1.8/0.90 | 1.9/0.40 | 1.3/0.32 | 3/0.46 |
| X-X-X-X-X-X-X-E | 12.6/6.4 | 15.2/8.2 | 11.5/6.0 | 13.5/6.0 |


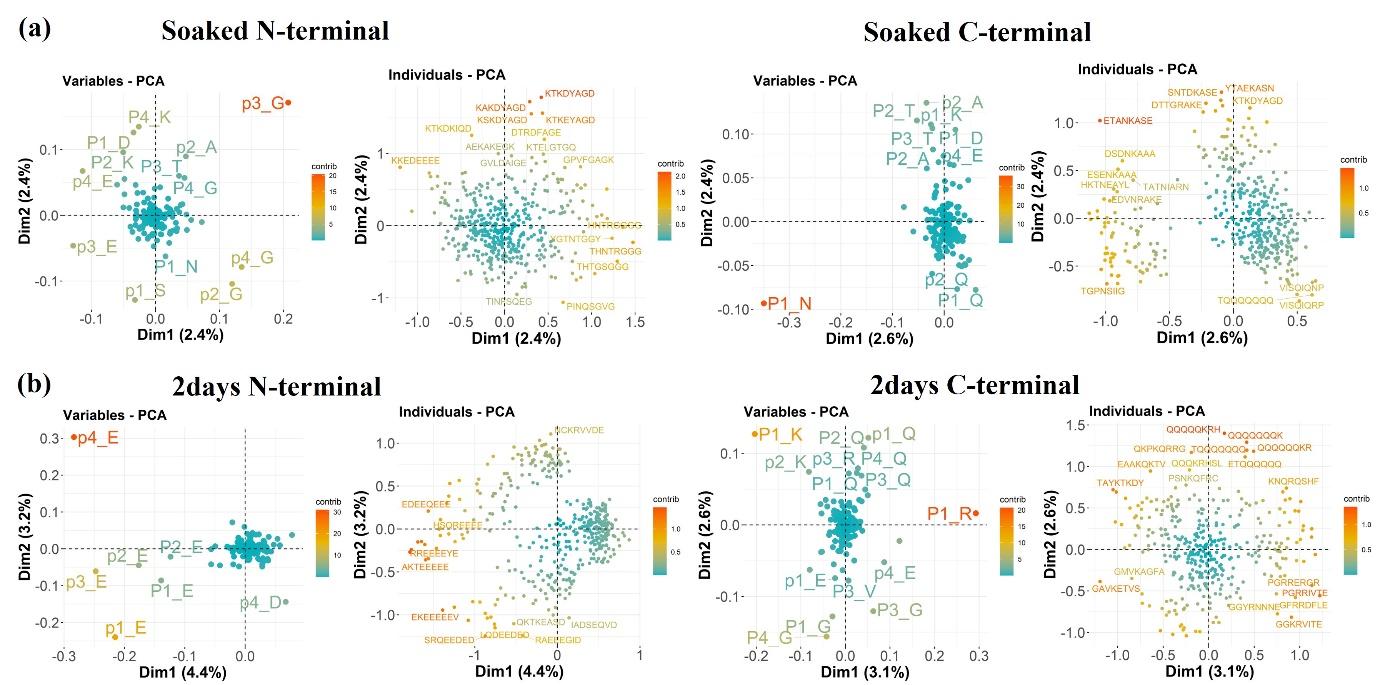


**Figure S5.** PCA plots showing amalgamation of distinct proteases preferring different substrates in brown chickpea (a) soaked and (b) 2 days samples

**Table S2:** Additional proteolytic site preferences in brown chickpea suggested from the principal component analysis represented as percentage of occurrence for N-terminal and C-terminal. Motifs are highlighted in bold. All numbers calculated as percentage of occurrence (no. of occurrence/no. of peptides)*100

| P4-P3-P2-P1-p1-p2-p3-p4 | Ungerminated  (N-term/C-term) | 2days  (N-term/C-term) | 4days  (N-term/C-term) | 6days  (N-term/C-term) |
| --- | --- | --- | --- | --- |
| X-X-X-X-X-X-G-X | 6.5/4.2 | 11.25/9.27 | 7.57/5.31 | 6.36/3.81 |
| X-X-X-D-X-X-X-X | 7.04/6.91 | 4.80/2.81 | 4.13/4.42 | 4.13/3.73 |
| X-X-X-X-X-G-X-G | 2.03/0.94 | 1.65/0.49 | 0.78/0.09 | 0.715/0 |
| X-X-X-X-X-X-X-E | 11.11/7.31 | 26.98/10.92 | 15.06/7.57 | 13.12/6.52 |
| X-X-X-X-E-X-E-X | 0.13/0.40 | 2.15/2.48 | 0.39/0.39 | 0.23/0.23 |
| X-X-X-K-X-X-X-X | 2.7/5.14 | 1.98/8.44 | 3.64/6.88 | 4.05/7.00 |
| X-X-Q-X-Q-X-X-X | 0.54/0.94 | 0.49/2.31 | 3.05/2.46 | 1.27/1.51 |
| G-X-X-G-X-X-X-X | 0.40/0.40 | 1.49/2.81 | 0.78/0.49 | 0.32/0.08 |
| X-X-X-R-X-X-X-X | 5.14/6.50 | 11.92/23.34 | 9.15/11.61 | 5.56/9.46 |
| X-X-G-X-X-X-X-E | 0.27/0.13 | 0.99/0.16 | 0.39/0 | 0.55/0.31 |
| X-X-X-K-X-X-X-X | 4.74/8.94 | 10.43/17.71 | 8.66/15.45 | 10.10/12.80 |
| X-X-X-N-X-X-X-X | 9.34/18.67 | 8.27/7.61 | 8.07/11.90 | 8.51/11.69 |
| X-X-X-X-A-X-X-X | 8.40/5.69 | 5.13/7.45 | 7.67/6.89 | 6.12/6.68 |
| X-X-X-X-D-X-X-X | 4.06/3.25 | 11.42/6.95 | 4.52/4.82 | 9.78/9.62 |
| X-X-X-Q-X-X-X-X | 5.55/11.11 | 4.13/6.29 | 4.72/11.02 | 9.22/12.09 |
| X-X-X-K-D-E-E-E | 0/0.40 | 1.49/1.32 | 0.29/0.68 | 1.59/2.06 |
| X-X-X-N-A-X-X-E | 0.27/1.08 | 0.662/0.49 | 0.59/0.88 | 0.71/1.13 |


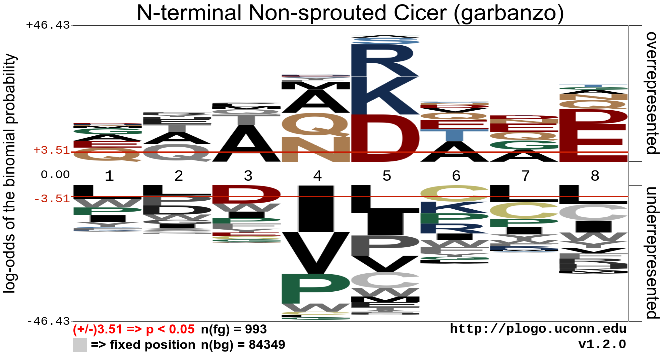

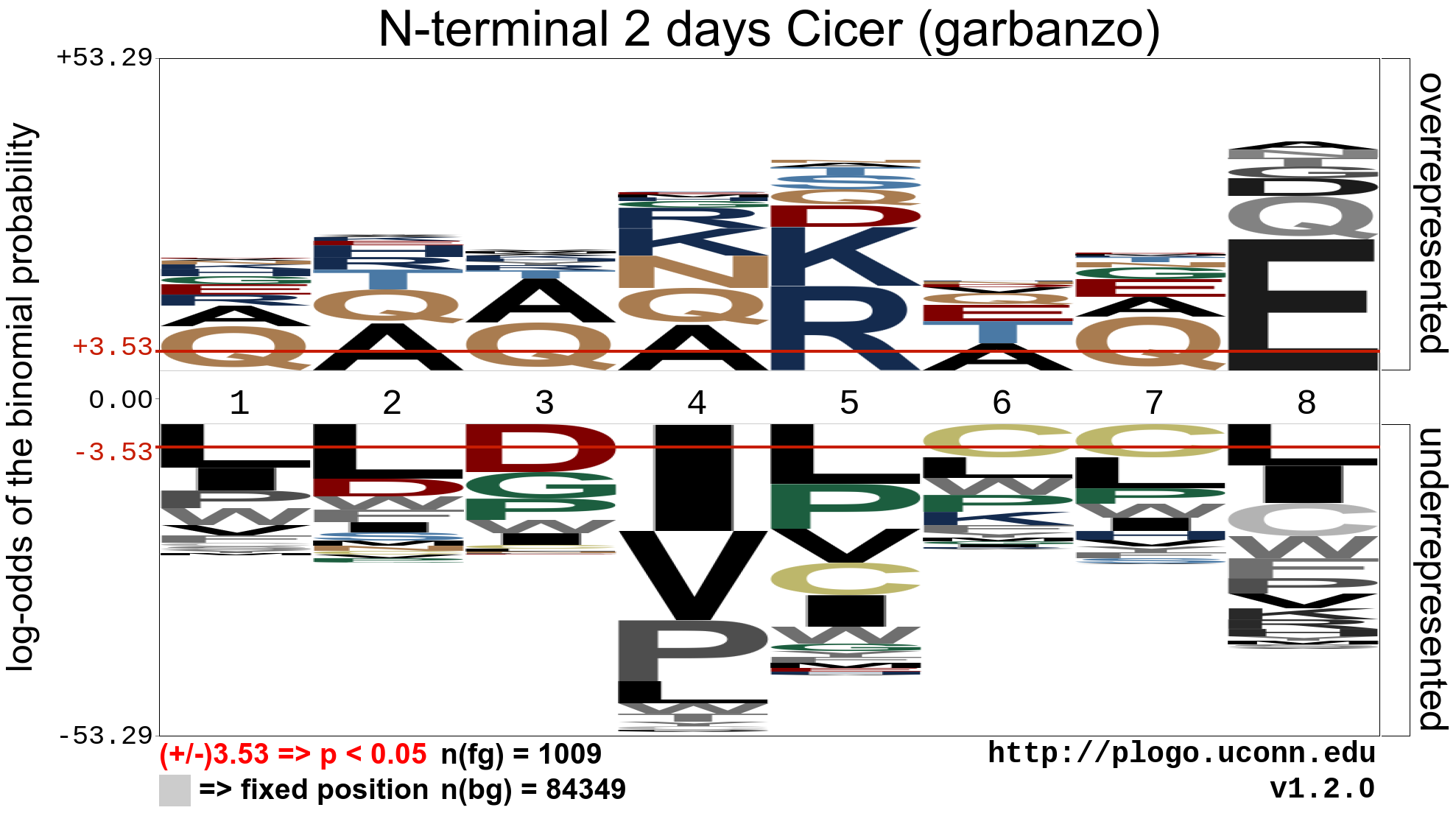

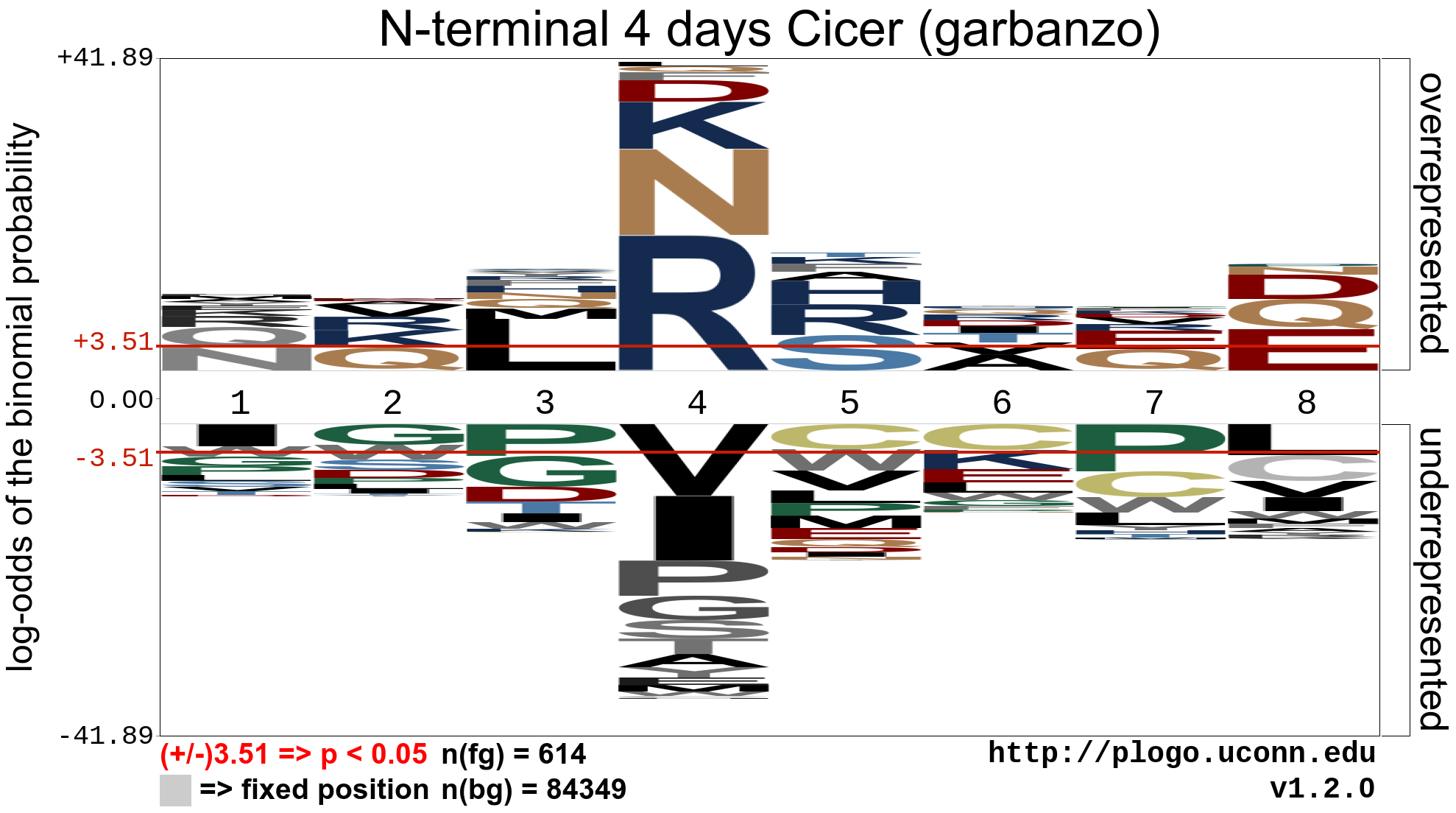

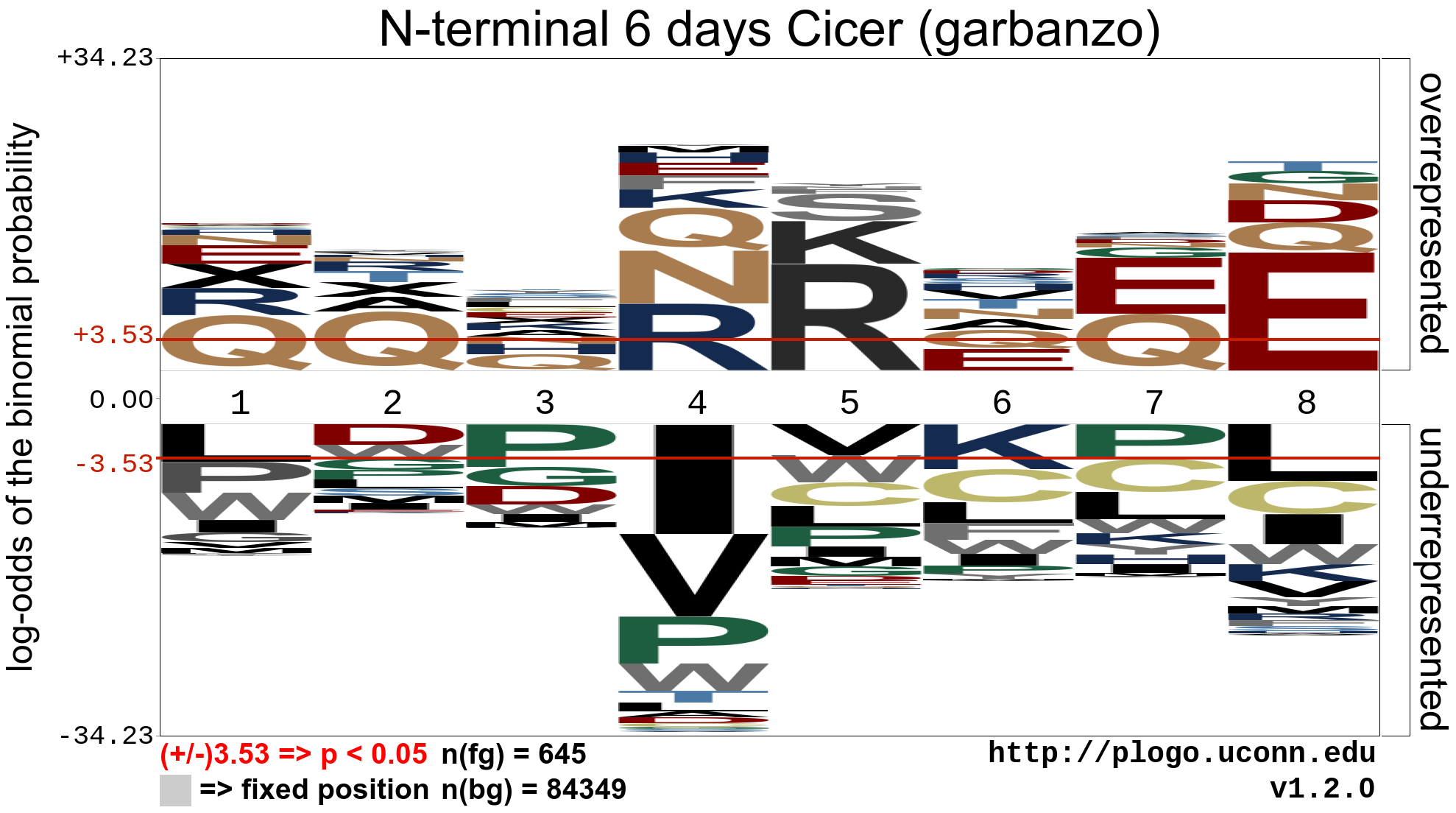

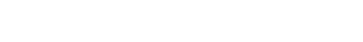

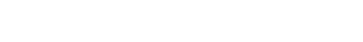

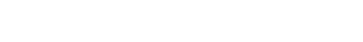

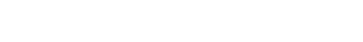

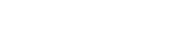

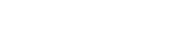

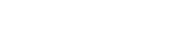

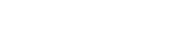

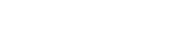

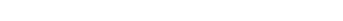

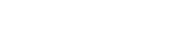

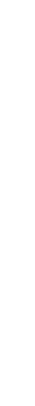

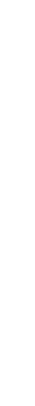

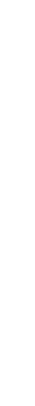

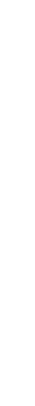

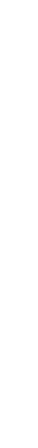

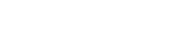

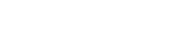

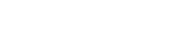

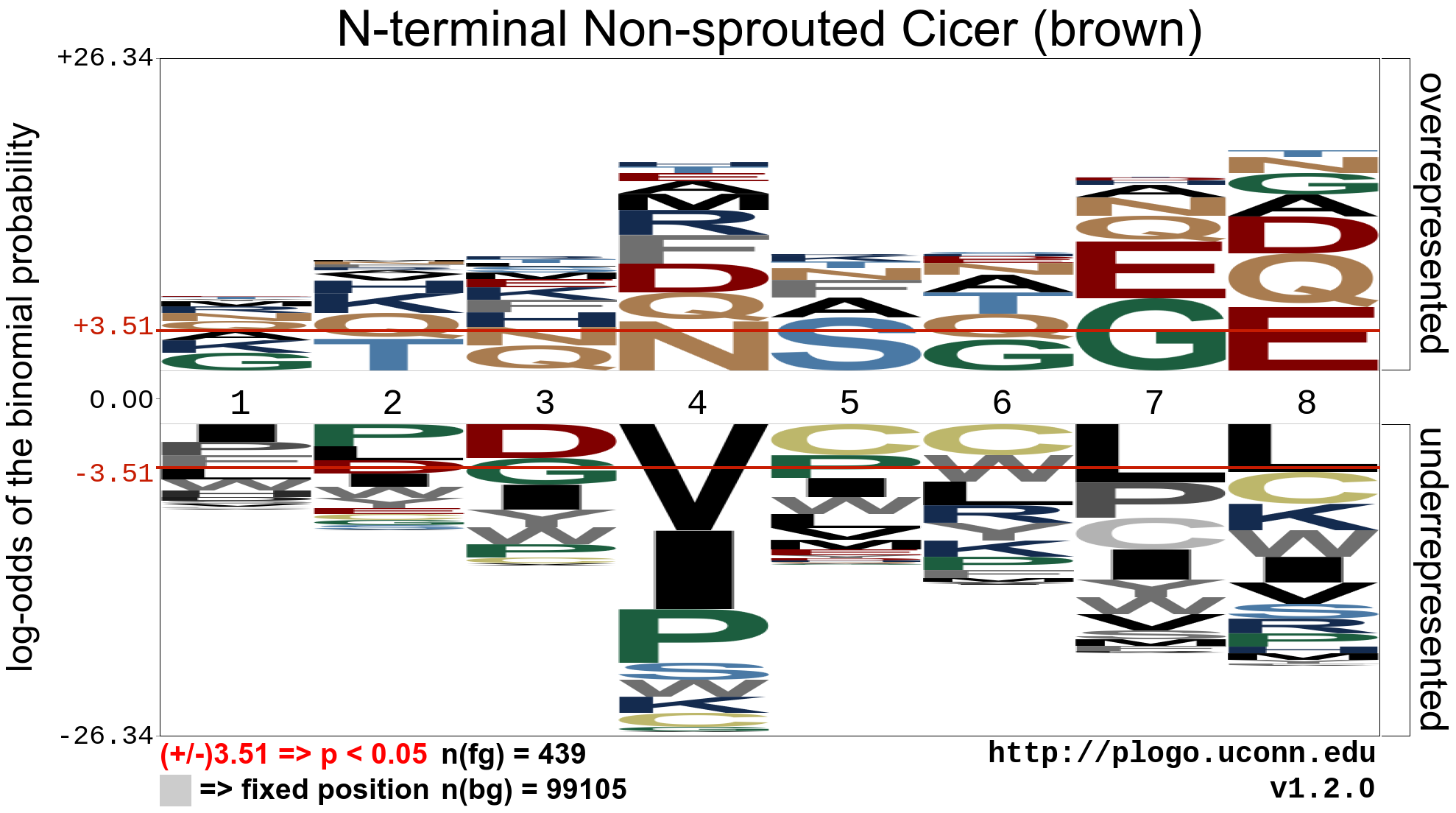

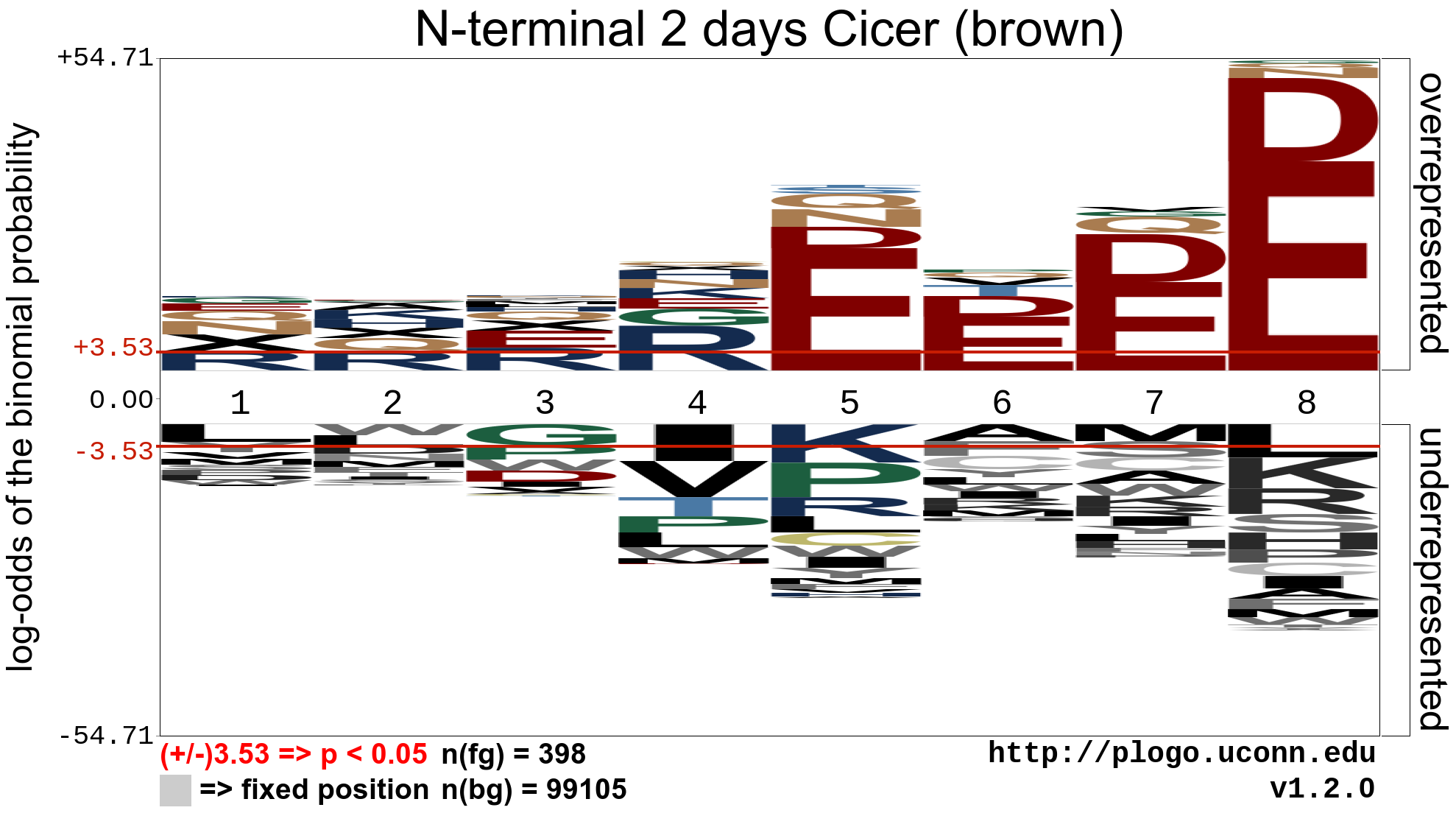

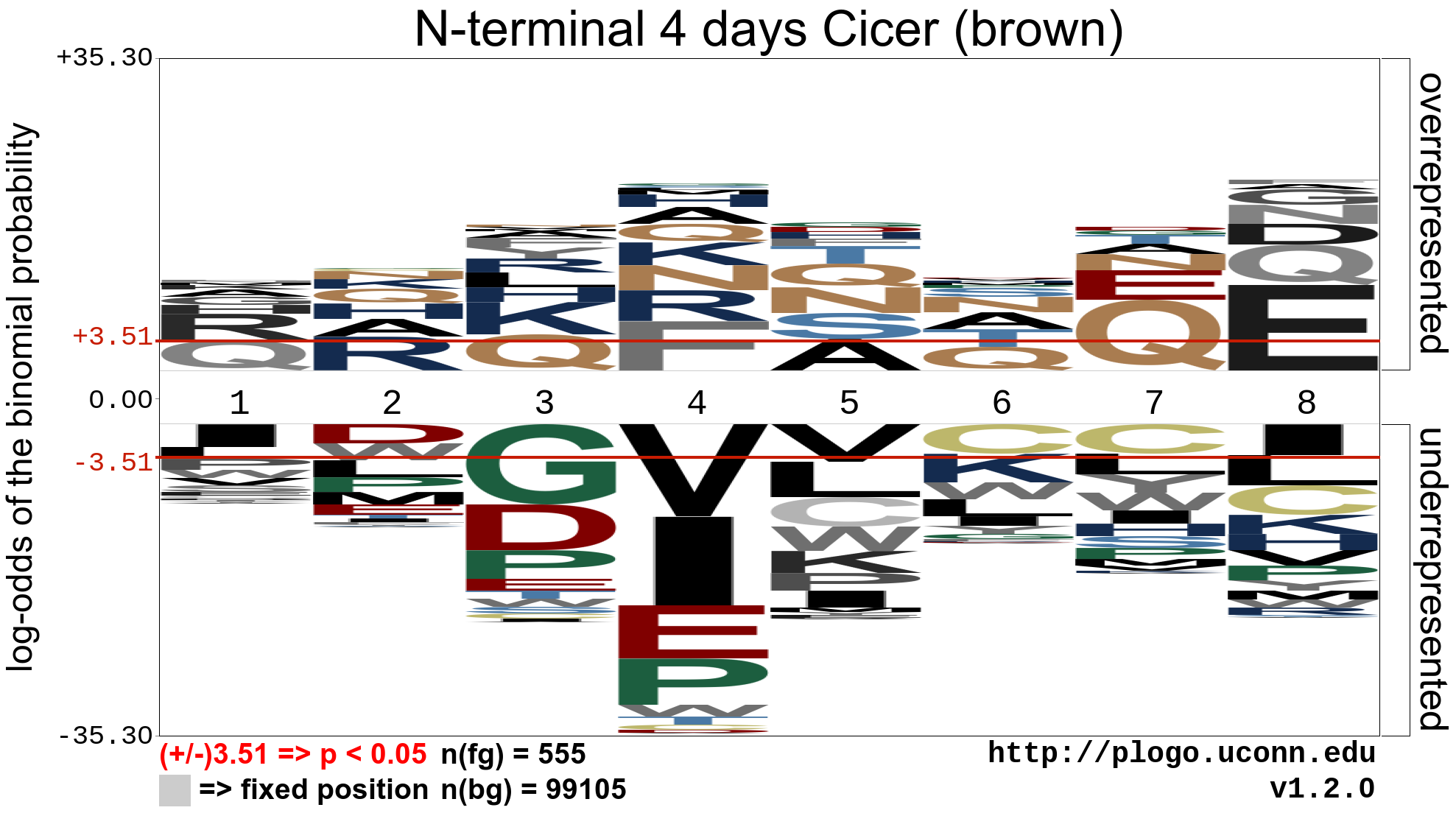

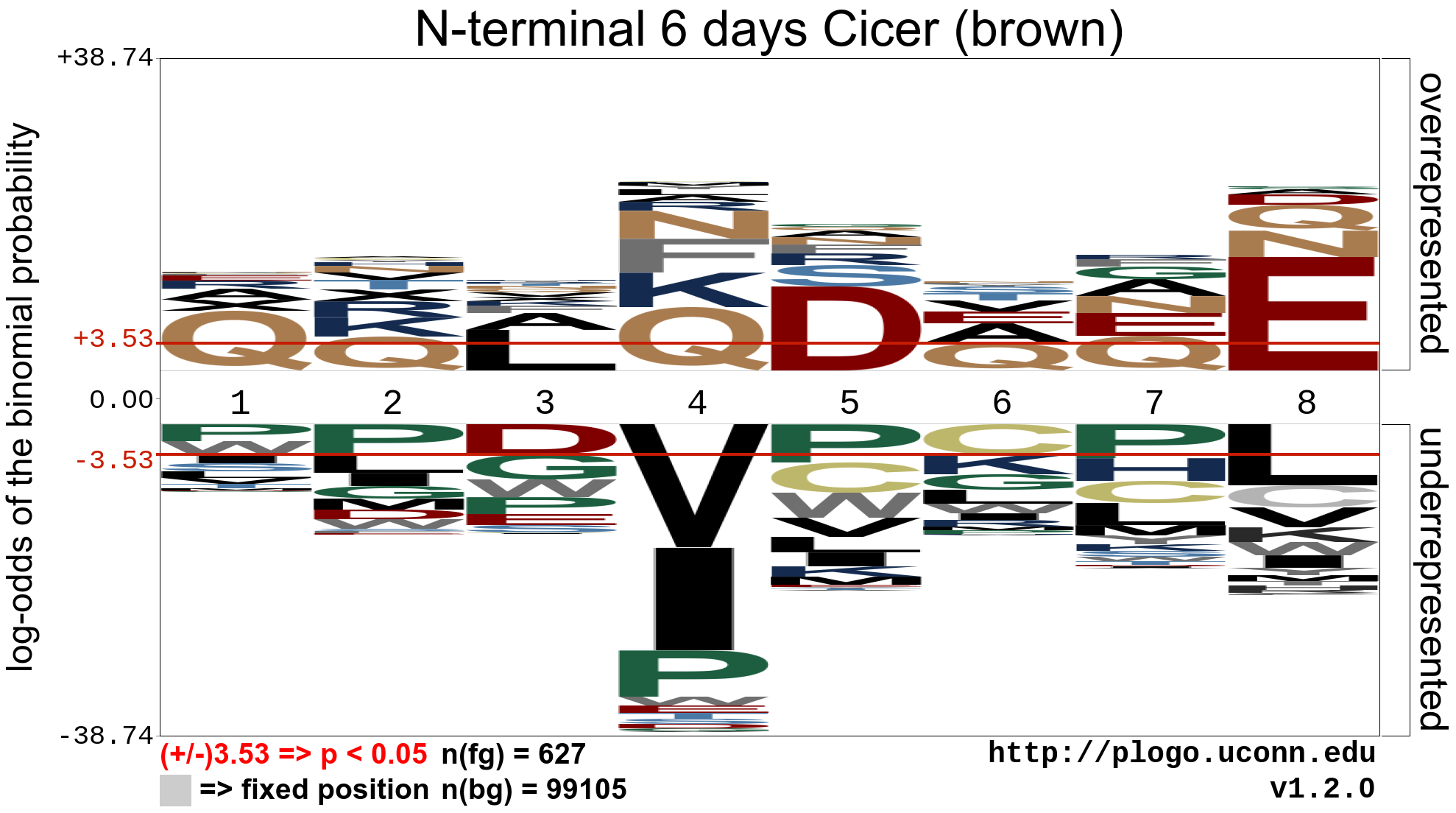

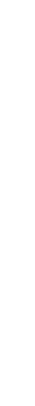

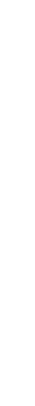

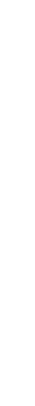

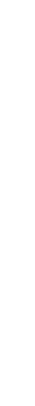

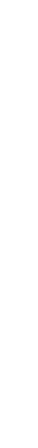

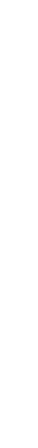

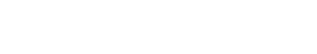

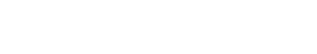

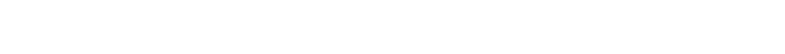

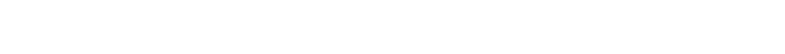


**Figure S6.** Sequence logos garbanzo and brown chickpea for N-terminal at different germination stages

**Table** **S3**: Evaluating how positively charged residue preferences at P1 and P1’ sites are co-distributed among N and C-terminal peptide cleavages in garbanzo. Odds ratio represents the enrichment of positively charged residues co-occurring at both P1 and P1’ sites.

| N-term | P1: R or K  P1’: R or K | P1: R or K  P1’: ≠R &≠ K | P1: ≠R & ≠K  P1’: R or K | P1: ≠R & ≠K  P1’: ≠R & ≠K | Odds Ratio | P-value |
| --- | --- | --- | --- | --- | --- | --- |
| Soaked | 43 | 163 | 377 | 1191 | 0.833 | 0.338 |
| 2 day | 53 | 311 | 371 | 1249 | 0.573 | 0.0003 |
| 4 day | 60 | 197 | 97 | 576 | 1.80 | 0.0016 |
| 6 day | 54 | 180 | 193 | 647 | 1.005 | 1 |

| C-term | P1: R or K  P1’: R or K | P1: R or K  P1’: ≠R &≠ K | P1: ≠R & ≠K  P1’: R or K | P1: ≠R & ≠K  P1’: ≠R& ≠K | Odds Ratio | P-value |
| --- | --- | --- | --- | --- | --- | --- |
| Soaked | 75 | 196 | 458 | 1045 | 0.873 | 0.3879 |
| 2 day | 88 | 390 | 486 | 1020 | 0.473 | 2.168e-09 |
| 4 day | 104 | 288 | 82 | 456 | 2.008 | 3.039e-05 |
| 6 day | 99 | 232 | 241 | 502 | 0.888 | 0.439 |

**Table** **S4**: Evaluating how positively charged residue preferences at P1 and P1’ sites are co-distributed among N and C-terminal peptide cleavages in brown chickpea.

| N-term | P1: R or K  P1’: R or K | P1: R or K  P1’: ≠R &≠ K | P1: ≠R & ≠K  P1’: R or K | P1: ≠R & ≠K  P1’: ≠R & ≠K | Odds Ratio | P-value |
| --- | --- | --- | --- | --- | --- | --- |
| Soaked | 4 | 69 | 67 | 598 | 0.517 | 0.29 |
| 2 day | 3 | 132 | 13 | 456 | 0.797 | 1 |
| 4 day | 14 | 167 | 60 | 775 | 1.08 | 0.75 |
| 6 day | 21 | 176 | 123 | 937 | 0.90 | 0.80 |

| C-term | P1: R or K  P1’: R or K | P1: R or K  P1’: ≠R &≠ K | P1: ≠R & ≠K  P1’: R or K | P1: ≠R & ≠K  P1’: ≠R& ≠K | Odds Ratio | P-value |
| --- | --- | --- | --- | --- | --- | --- |
| Soaked | 26 | 88 | 129 | 495 | 1.13 | 0.617 |
| 2 day | 17 | 231 | 21 | 335 | 1.17 | 0.733 |
| 4 day | 28 | 247 | 96 | 645 | 0.76 | 0.28 |
| 6 day | 49 | 231 | 156 | 821 | 1.11 | 0.58 |


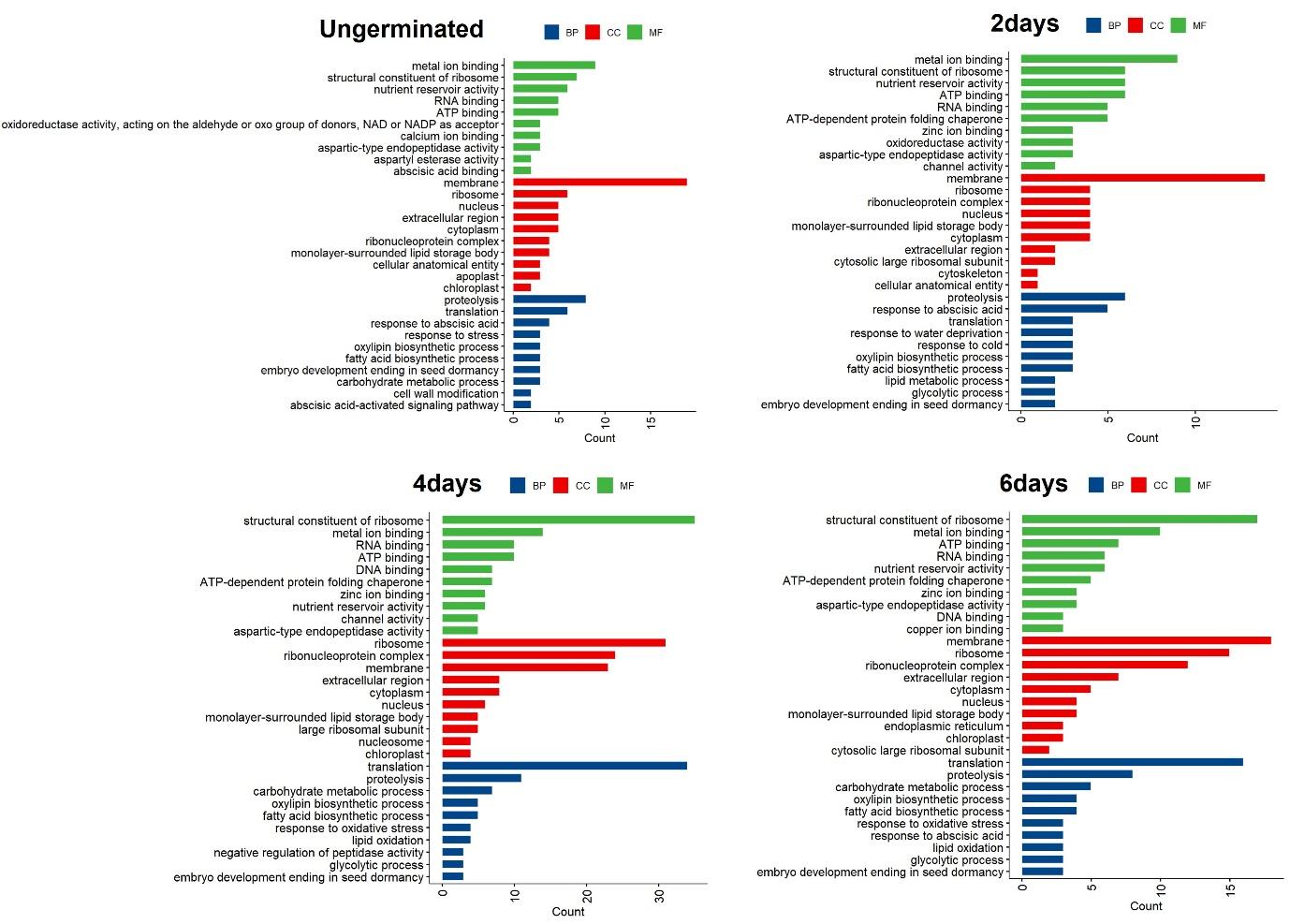


**Figure S7.** Top 8 most significantly enriched GO classes with reference to the chickpea proteome; from non-trypsinised samples BP -Biological process, CC – Cellular component, MF- Molecular function (GO was calculated using GeneOntologyObj function of UniprotR package)


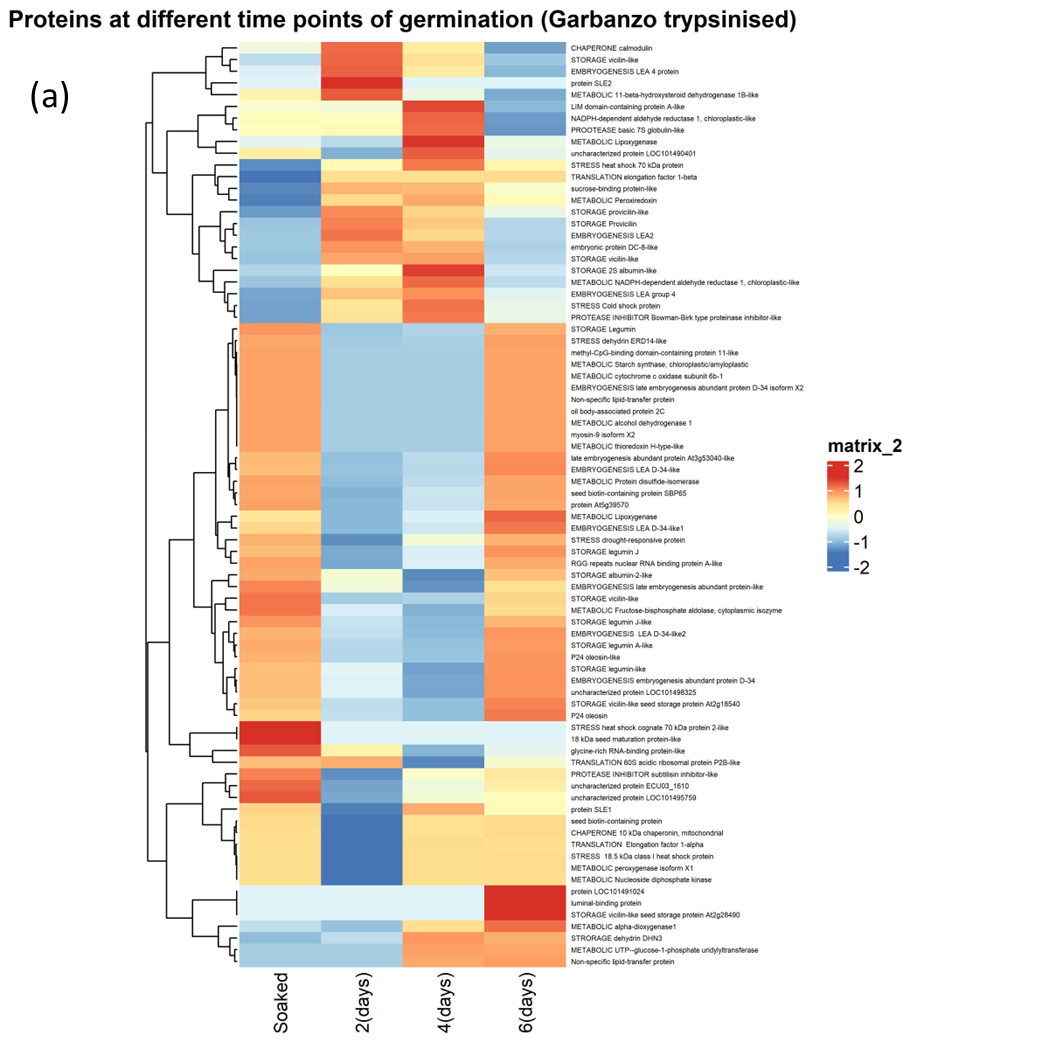


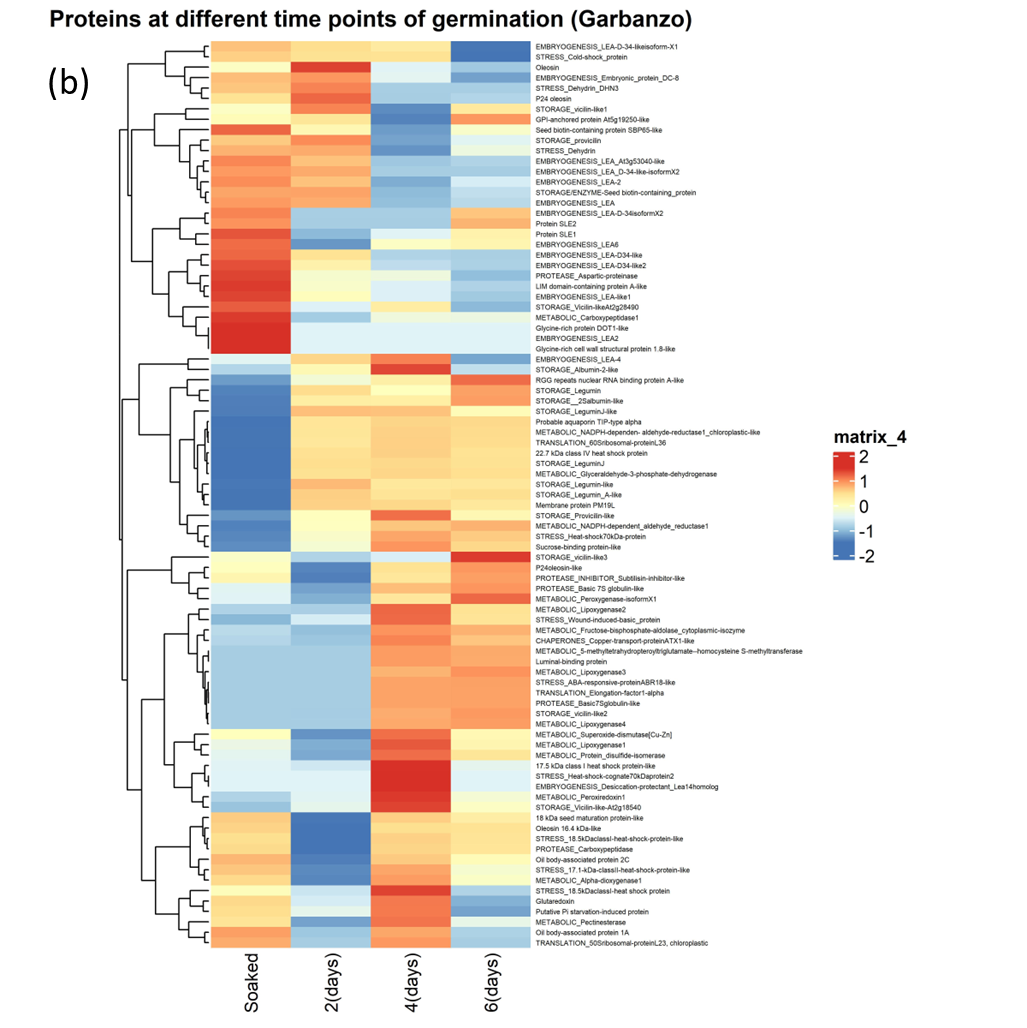


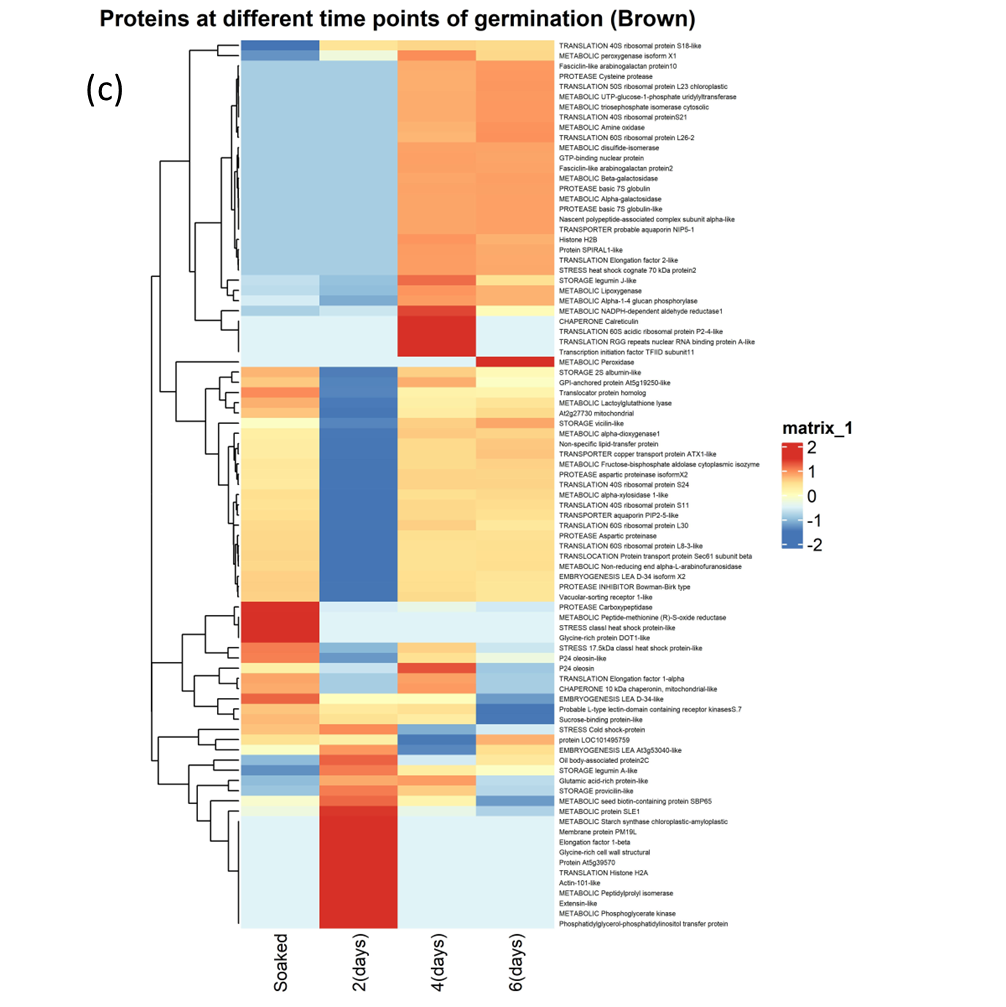


**Figure S8.** Heatmap of z-scores of log10(intensity) of top 100 proteins in (a) garbanzo trypsinized (b) non-trypsinised garbanzo and (c) non-trypsinised brown chickpea

**Table S5.** Peptide counts and % for different classes of proteins at different germination time points for all replicates.

| GARBANZO | S1 | S2 | S3 | 2.1 | 2.2 | 2.3 |
| --- | --- | --- | --- | --- | --- | --- |
| Storage | 877/49.68 | 1885/39.33 | 1350/43.29 | 2393/52.18 | 1949/45.78 | 736/54.47 |
| Embryogenesis | 475/26.91 | 1461/30.48 | 857/27.48 | 1062/23.15 | 1232/28.94 | 210/15.54 |
| Protease | 24/1.36 | 87/1.82 | 61/1.96 | 45/.98 | 35/.82 | 22/1.63 |
| Protease inhibitors | 7/.4 | 15/.31 | 8/.26 | 9/.2 | 3/.07 | 6/.44 |
| Chaperones | 1/.06 | 5/.10 | 2/.06 | 3/.06 | 3/.07 | 2/.15 |
| Stress | 121/6.85 | 397/8.28 | 250/8.01 | 303/6.60 | 318/7.47 | 158/11.69 |
| Metabolic | 85/4.81 | 310/6.46 | 215/6.89 | 181/3.94 | 170/3.99 | 114/8.43 |
| Translation | 1/.06 | 3/.06 | 2/.06 | 4/.09 | 4/.09 | 2/.15 |

| GARBANZO | 4.1 | 4.2 | 4.3 | 6.1 | 6.2 | 6.3 |
| --- | --- | --- | --- | --- | --- | --- |
| Storage | 937/53.60 | 1039/50.73 | 1206/50.71 | 2199/64.52 | 3602/59.80 | 2041/57.27 |
| Embryogenesis | 163/9.32 | 202/9.86 | 243/10.21 | 150/6.82 | 406/11.27 | 223/10.92 |
| Protease | 60/3.43 | 65/3.17 | 70/2.94 | 59/2.68 | 99/2.75 | 74/3.63 |
| Protease inhibitors | 6/.34 | 8/.39 | 10/.42 | 7/.32 | 9/.25 | 9/.44 |
| Chaperones | 3/.17 | 4/.19 | 5/.21 | 4/.18 | 6/.17 | 3/.15 |
| Stress | 197/11.27 | 201/9.81 | 253/10.63 | 181/8.23 | 285/7.91 | 165/8.08 |
| Metabolic | 231/13.21 | 326/15.91 | 356/14.97 | 219/9.95 | 289/8.02 | 225/11.02 |
| Translation | 13/.74 | 20/.98 | 23/.97 | 5/.22 | 14/.39 | 6/.29 |

**Table S6.** Peptide counts and % for different classes of proteins at different germination time points (Brown non-trpysinised) for all replicates

| BROWN | S1 | S2 | S3 | 2.1 | 2.2 | 2.3 |
| --- | --- | --- | --- | --- | --- | --- |
| Storage | 1338/58.38 | 1493/58.27 | 1552/55.39 | 976/56.42 | 875/55.27 | 996/55.67 |
| Embryogenesis | 468/20.42 | 512/19.98 | 636/22.7 | 338/19.54 | 301/19.01 | 333/18.61 |
| Protease inhibitors | 60/2.62 | 70/2.73 | 71/2.53 | 12/.69 | 12/.76 | 21/1.17 |
| Stress | 126/5.5 | 140/5.46 | 157/5.6 | 83/4.8 | 69/4.36 | 83/4.64 |
| Metabolic | 155/6.76 | 186/7.26 | 191/6.82 | 134/7.75 | 140/8.84 | 149/8.33 |
| Translation | 2/.087 | 3/.12 | 0/0 | 5/.29 | 4/.25 | 6/.34 |

| BROWN | 4.1 | 4.2 | 4.3 | 6.1 | 6.2 | 6.3 |
| --- | --- | --- | --- | --- | --- | --- |
| Storage | 1893/62.89 | 1897/59.86 | 1901/64.15 | 2568/70.09 | 2739/65.9 | 2561/70.65 |
| Embryogenesis | 321/10.66 | 398/12.56 | 302/10.19 | 333/9.09 | 444/10.68 | 329/9.07 |
| Protease inhibitors | 103/3.42 | 117/3.69 | 92/3.1 | 130/3.54 | 155/3.73 | 135/3.72 |
| Stress | 126/4.19 | 157/4.95 | 144/4.86 | 128/3.49 | 154/3.71 | 108/2.98 |
| Metabolic | 366/12.16 | 370/11.68 | 345/11.64 | 354/9.66 | 492/11.84 | 353/9.74 |
| Translation | 3/.99 | 2/.063 | 2/.067 | 4/.11 | 1/.024 | 1/.027 |

**Table S7.** Peptide counts and % for different classes of proteins at different germination time points (Garbanzo trpysinised) for all replicates

| GARBANZO | S1 | S2 | S3 | 2.1 | 2.2 | 2.3 |
| --- | --- | --- | --- | --- | --- | --- |
| Storage | 217/43.06 | 253/42.17 | 257/42.2 | 277/40.74 | 254/38.02 | 269/37.36 |
| Embryogenesis | 107/21.23 | 121/20.17 | 119/19.54 | 146/21.47 | 138/20.66 | 145/20.14 |
| Protease inhibitors | 7/1.39 | 7/1.17 | 6/.98 | 6/.88 | 6/.90 | 5/.69 |
| Chaperones | 10/1.98 | 14/2.33 | 16/2.63 | 18/2.64 | 12/1.796 | 13/1.8 |
| Stress | 17/3.37 | 19/3.17 | 22/3.61 | 30/4.41 | 32/4.79 | 35/4.86 |
| Metabolic | 35/6.94 | 49/8.17 | 49/8.04 | 61/8.97 | 68/10.18 | 75/10.41 |
| Translation | 5/.99 | 9/1.15 | 8/1.31 | 11/1.61 | 11/1.64 | 15/2.08 |

| GARBANZO | 4.1 | 4.2 | 4.3 | 6.1 | 6.2 | 6.3 |
| --- | --- | --- | --- | --- | --- | --- |
| Storage | 320/36.49 | 325/36.59 | 311/35.83 | 262/39.16 | 275/40.68 | 234/39.59 |
| Embryogenesis | 150/17.1 | 165/18.58 | 169/19.47 | 154/23.02 | 146/21.6 | 133/22.5 |
| Protease inhibitors | 7/.80 | 7/.79 | 8/.92 | 8/1.19 | 6/.88 | 6/1.01 |
| Chaperones | 26/2.96 | 28/3.15 | 23/2.65 | 22/3.29 | 14/2.07 | 12/2.03 |
| Stress | 49/5.59 | 46/5.18 | 49/5.65 | 32/4.78 | 39/5.77 | 32/5.41 |
| Metabolic | 112/12.77 | 113/12.73 | 101/11.64 | 38/5.68 | 41/6.06 | 32/6.41 |
| Translation | 20/2.28 | 17/1.91 | 16/1.84 | 7/1.04 | 10/1.48 | 7/1.18 |

**Table S8** Average peptide count of protease and protease inhibitors at 4 time points for Cicer (Garbanzo) , non-tryptic samples

| **Protease/Inhibitor** | **0 day** | **2 days** | **4 days** | **6 days** |
| --- | --- | --- | --- | --- |
| Aspartic proteinase | 5 | 1 | 2.67 | 1.33 |
| Subtilase | 0 | 0.33 | 0 | 0 |
| Cysteine proteinase | 0 | 0 | 0.33 | 0.33 |
| Basic 7S globulin-like | 0.33 | 0.33 | 0 | 0 |
| Lysosomal Pro-X carboxypeptidase isoform X1 | 0.67 | 0.33 | 0 | 0.33 |
| Aspartic proteinase NANA, chloroplast | 0 | 0 | 0.33 | 0 |
| Ubiquitin carboxyl-terminal hydrolase | 0 | 0.33 | 0 | 0 |
| Ubiquitin carboxyl-terminal hydrolase 15 | 0 | 0 | 0 | 0.33 |
| Bowman-Birk type proteinase inhibitor-like | 0.67 | 0.33 | 0.33 | 0.67 |
| Carboxypeptidase | 0 | 0 | 0 | 0.33 |
| Probable zinc metalloprotease EGY1, chloroplastic | 0.33 | 0.33 | 0 | 0 |
| Insulin-degrading enzyme-like 1, peroxisomal isoform X1 GN | 0 | 0 | 1 | 0 |
| Aspartic proteinase like | 2.67 | 0.33 | 2.33 | 1.33 |
| Cathepsin B-like protease 2 | 0 | 0 | 0 | 0.33 |
| Basic 7S globulin | 0 | 0.33 | 1 | 0 |
| Disease resistance RPP13-like protein 2 | 0 | 0 | 0 | 0.33 |
| Subtilisin-like protease SBT1.3 | 1.33 | 0 | 1.33 | 0 |
| Probable cysteine protease RD21B | 0.33 | 0 | 0 | 0 |
| Vacuolar-sorting receptor 1-like | 1.33 | 1 | 0.67 | 1 |
| Aspartyl protease AED3-like | 0 | 0 | 1.67 | 0.33 |
| Vacuolar-processing enzyme-like isoform X1 | 0.67 | 0.33 | 0 | 0.33 |
| Ubiquitin carboxyl-terminal hydrolase 18-like | 0.67 | 0.33 | 0.33 | 0 |
| Subtilisin inhibitor-like | 6.33 | 2 | 4.33 | 6 |
| ATP-dependent zinc metalloprotease FTSH, chloroplastic | 0 | 0.33 | 0 | 0 |
| CO(2)-response secreted protease-like | 0.67 | 0 | 0 | 0 |
| Aminopeptidase | 0 | 0 | 0.67 | 0 |
| Cysteine proteinase inhibitor B | 0 | 2 | 3 | 3 |
| Aspartic proteinase nepenthesin-1 | 0 | 0.33 | 0 | 0 |
| Cysteine proteinase inhibitor 4-like | 0.33 | 0 | 0.67 | 0.67 |
| Thiol protease aleurain-like | 0 | 0 | 0 | 0.33 |
| Vacuolar-sorting receptor 1 | 0 | 0.33 | 0.67 | 0.33 |
| Subtilisin-like protease SBT1.7 | 0.33 | 0 | 0.33 | 0 |
| Proteasome | 0.33 | 0 | 0.33 | 0 |
| Carboxypeptidase (Fragment) | 2.67 | 0.67 | 2.67 | 1 |
| Legumain | 4.33 | 0.33 | 0 | 0.33 |
| Cysteine proteinase inhibitor 5-like | 0.33 | 0 | 0 | 0 |
| Disease resistance protein RGA1 | 0 | 0 | 0.67 | 0 |
| ADP-ribosyl cyclase/cyclic ADP-ribose hydrolase | 0 | 0 | 0.67 | 0 |
| ADP-ribosyl cyclase/cyclic ADP-ribose hydrolase SV=2 | 0 | 0 | 0.33 | 0.33 |
| TMV resistance protein N-like isoform X1 | 0.33 | 0 | 0 | 0 |
| Cucumisin-like | 0.67 | 0.67 | 0 | 0 |
| Disease resistance RPP13-like protein 1 | 0.33 | 1 | 0.33 | 0.67 |
| TMV resistance protein N-like | 0.33 | 0 | 0 | 0 |
| Subtilisin-like protease SBT6.1 | 0 | 0 | 0 | 0.33 |
| Putative Pi starvation-induced protein | 2 | 1 | 3.67 | 0.67 |
| Gag-protease polyprotein-like protein (Fragment) | 0 | 0 | 0 | 0.33 |
| NBS-LRR protein | 0 | 0 | 0.33 | 0 |

**Table S9** Average peptide count of protease and protease inhibitors at 4 time points for Cicer (Brown) non-tryptic samples

| **Protease/Inhibitor** | **0 day** | **2 days** | **4 days** | **6 days** |
| --- | --- | --- | --- | --- |
| Aspartic proteinase | 7.33 | 0.33 | 2.67 | 2 |
| Cysteine proteinase | 0 | 0 | 0.33 | 1 |
| Protease Do-like 8, chloroplastic | 0 | 0.33 | 0.33 | 0 |
| Carboxypeptidase | 0.67 | 0 | 0 | 1 |
| Serpin-ZX-like | 0 | 0.33 | 0 | 0 |
| Bowman-Birk type proteinase inhibitor-like | 1 | 0 | 1.33 | 1.33 |
| Insulin-degrading enzyme-like 1, peroxisomal isoform X1 GN | 0.67 | 0 | 0 | 1.67 |
| Aspartic protease like | 1 | 0 | 1.33 | 3 |
| Basic 7S globulin-like | 12 | 10.33 | 26 | 57.67 |
| Subtilisin-like protease SBT1.8 | 0 | 0 | 0.33 | 0 |
| Basic 7S globulin | 0 | 0 | 0.67 | 0.67 |
| Vacuolar-sorting receptor 1-like | 0.33 | 0 | 0 | 0.33 |
| Aspartyl protease AED3-like | 0 | 0.33 | 0 | 0 |
| Vacuolar-processing enzyme-like isoform X1 | 1 | 0 | 0 | 0 |
| ATP-dependent zinc metalloprotease FTSH 11, chloroplastic/mitochondrial | 0 | 0.33 | 0 | 0 |
| Ubiquitin carboxyl-terminal hydrolase 18-like | 0 | 0.33 | 0.33 | 1.33 |
| Probable disease resistance protein At1g61180 GN=LOC1015 | 0.33 | 0.33 | 0 | 0 |
| Mitochondrial intermediate peptidase, mitochondrial isoform X1 | 0.33 | 0 | 0 | 0 |
| Subtilisin inhibitor-like | 2 | 0.33 | 3.67 | 6.67 |
| ATP-dependent zinc metalloprotease FTSH, chloroplastic | 0.33 | 0 | 0 | 0 |
| CO(2)-response secreted protease-like | 0.33 | 0 | 0 | 0 |
| Cysteine proteinase inhibitor B | 0 | 0.33 | 3.67 | 9 |
| Thylakoidal processing peptidase 1, chloroplastic | 0.67 | 0 | 0 | 0 |
| Aspartic proteinase Asp1-like | 0 | 0 | 0 | 0.33 |
| Cysteine proteinase inhibitor 4-like | 0 | 0.33 | 0 | 0.33 |
| Zingipain-2-like | 0 | 0 | 0 | 0.67 |
| Subtilisin-like protease SBT5.3 | 0 | 0 | 0.33 | 0 |
| TMV resistance protein N isoform X2 | 0.33 | 0 | 0 | 0 |
| Thiol protease aleurain-like | 0 | 0 | 0 | 0.33 |
| Subtilisin-like protease SBT1.4 | 0 | 0 | 0.33 | 0 |
| Methionine aminopeptidase | 0.33 | 0 | 0 | 0 |
| Serine carboxypeptidase-like 13 isoform X1 | 0 | 0.33 | 0 | 0 |
| Carboxypeptidase (Fragment) | 4 | 0.67 | 0 | 1.67 |
| TMV resistance protein N-like | 0 | 0.33 | 0 | 0 |
| Metalloendoproteinase 5-MMP-like | 0 | 0 | 0 | 0.33 |
| Legumain | 1 | 0 | 0 | 0 |
| Metalloendoproteinase 1-like | 0 | 0 | 0.33 | 0 |
| ATP-dependent zinc metalloprotease FTSH 10, mitochondrial-like | 0 | 0.33 | 0 | 0 |
| Aspartic proteinase-like protein 2 | 0 | 0 | 0 | 0.33 |
| ADP-ribosyl cyclase/cyclic ADP-ribose hydrolase SV=2 | 0.33 | 0 | 0 | 0 |
| TMV resistance protein N-like isoform X1 | 0.33 | 0 | 0 | 0.33 |
| Disease resistance protein At3g14460 | 0 | 0 | 0 | 0.33 |
| Putative Pi starvation-induced protein | 0.33 | 0 | 0.67 | 1.67 |
| CC-NBS-LRR disease resistance protein | 0 | 0.33 | 0 | 0 |

**Table S10.** Average count of protease and protease inhibitors protein identifications at 4 time points for Cicer (Garbanzo) from tryptic samples

| **Protease_inhibitor** | **0 day** | **2 days** | **4 days** | **6 days** |
| --- | --- | --- | --- | --- |
| Lysosomal Pro-X carboxypeptidase | 0 | 0.67 | 0 | 0.33 |
| CC-NBS-LRR disease resistance protein | 0 | 0.33 | 0 | 0 |
| ADP-ribosyl cyclase/cyclic ADP-ribose hydrolase | 0 | 0 | 0.67 | 0 |
| Basic 7S globulin-like | 0 | 0 | 0 | 0.67 |
| Ubiquitin carboxyl-terminal hydrolase | 0.33 | 0 | 0 | 0.33 |
| Disease resistance protein At1g50180 | 0 | 0 | 0 | 0.33 |
| Ubiquitin carboxyl-terminal hydrolase | 0 | 0 | 0 | 0.33 |
| Bowman-Birk type proteinase inhibitor-like | 4 | 4.67 | 5 | 4.67 |
| Cysteine protease | 0 | 0.33 | 0 | 0 |
| Vacuolar-sorting receptor 1-like | 0 | 0 | 0.67 | 0.67 |
| Probable zinc metalloprotease EGY1, chloroplastic | 0.33 | 0 | 0 | 0.33 |
| Ubiquitin carboxyl-terminal hydrolase | 0 | 0 | 0 | 0.33 |
| Basic 7S globulin-like | 3.33 | 4.67 | 3.33 | 1.33 |
| Probable inactive ATP-dependent zinc metalloprotease FTSHI 5, chloroplastic | 1 | 0 | 0 | 0 |
| Basic 7S globulin | 0.33 | 0.33 | 0.67 | 0.33 |
| Probable cysteine protease RD19D | 0 | 0 | 0.33 | 0 |
| Basic 7S globulin-like | 0 | 0 | 0 | 0.67 |
| Subtilisin-like protease SBT1.3 | 0.33 | 0 | 0 | 0 |
| Protein ASPARTIC PROTEASE IN GUARD CELL 1 | 0 | 0.33 | 0 | 0 |
| Spliceosome-associated protein 130 A | 0.33 | 0 | 0 | 0 |
| Vacuolar-sorting receptor 1-like | 0.33 | 0 | 0.67 | 0.67 |
| Protein ASPARTIC PROTEASE IN GUARD CELL 2 | 0 | 0 | 0.33 | 0 |
| WD repeat-containing protein PCN | 0.33 | 0 | 0 | 0 |
| Subtilisin inhibitor-like | 3.67 | 2.33 | 3.33 | 3 |
| Vacuolar-processing enzyme-like | 0 | 0 | 0 | 0.33 |
| Ubiquitin carboxyl-terminal hydrolase 17 isoform X1 GN=LOC1015 | 0.33 | 0 | 0 | 0.67 |
| Probable Ufm1-specific protease | 0 | 0 | 1 | 0 |
| Chaperone protein ClpD, chloroplastic | 0 | 0 | 0 | 0.33 |
| Puromycin-sensitive aminopeptidase | 0 | 0 | 0.33 | 0.33 |
| Probable inactive ATP-dependent zinc metalloprotease FTSHI 4, chloroplastic | 0.33 | 0 | 0.33 | 0.67 |
| Probable inactive ATP-dependent zinc metalloprotease FTSHI 1, chloroplastic | 0 | 0 | 0 | 0.33 |
| Calpain-type cysteine protease DEK1 | 0 | 0.33 | 0 | 0 |
| Subtilisin-like protease SBT5.3 | 0 | 0.33 | 0 | 0 |
| Disease resistance protein RPM1 | 0 | 0 | 0.33 | 0 |
| Presenilin | 0 | 0.33 | 0 | 0 |
| Lon protease homolog 2, peroxisomal | 0 | 0 | 0 | 0.33 |
| Subtilisin-like protease SBT1.7 | 0 | 0 | 0.33 | 0 |
| TMV resistance protein N isoform X1 | 0 | 0 | 0.67 | 0 |
| Disease resistance protein RML1A | 0 | 0.33 | 0 | 0 |
| Presequence protease 1, chloroplastic/mitochondrial-like | 1 | 1 | 1 | 0.33 |
| Vacuolar-sorting receptor 1 | 0.67 | 0 | 0.33 | 0.33 |
| Subtilisin-like protease SBT3.9 isoform X1 | 0.33 | 0 | 0 | 0 |
| ATP-dependent Clp protease adapter protein CLPS1, chloroplastic | 0 | 0.67 | 0.33 | 0 |
| TMV resistance protein N-like | 0 | 0.33 | 0.33 | 0 |
| Probable disease resistance protein At4g27220 isoform X1 | 0 | 0 | 0 | 0.33 |
| CO(2)-response secreted protease-like | 0.67 | 0 | 0 | 1 |
| Protein cereblon-like isoform X2 | 0.67 | 0.33 | 0.33 | 0 |
| Zingipain-2-like | 0 | 0 | 0 | 0.33 |
| CC-NBS-LRR disease resistance protein | 0.33 | 0 | 0 | 0 |
| CC-NBS-LRR disease resistance protein | 0 | 0 | 0.67 | 0 |
| Probable disease resistance protein At5g66900 | 0.33 | 0 | 0.67 | 0.33 |
| Cysteine proteinase inhibitor | 0.67 | 0.33 | 0.33 | 0.33 |
| TMV resistance protein N-like isoform X1 | 0 | 0 | 0 | 0.33 |
| LOW QUALITY PROTEIN: ubiquitin carboxyl-terminal hydrolase 26-like | 0 | 0 | 0.67 | 0.33 |
| Vacuolar-processing enzyme beta-isozyme-like | 0 | 1 | 0 | 0.33 |
| Aspartic proteinase-like protein 2 | 0 | 0 | 0.33 | 0 |
| Disease resistance protein At3g14460 | 0 | 0 | 0 | 0.33 |
| Putative Pi starvation-induced protein | 1.33 | 2.33 | 2.33 | 1.33 |
| Disease resistance RPP13-like protein 1 | 0 | 0 | 0.33 | 0 |
| CC-NBS-LRR disease resistance protein | 0 | 1 | 0.67 | 0.33 |
| CC-NBS-LRR disease resistance protein | 0.33 | 0.67 | 1 | 0.33 |
