# Supplementary figures and images for: Motif mapping during chickpea germination reveals a complex sequential activation of different proteolytic activities
Source: PLoS One. 2024 Oct 31;19(10):e0307481. doi: 10.1371/journal.pone.0307481 (PMC11527212; doi:10.1371/journal.pone.0307481)

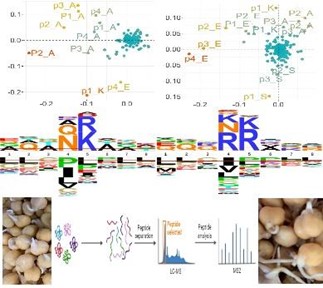

Supplement: S3 File — (JPG) [file pone.0307481.s003.jpg]
